# Exploring the Potential of Site-Selective Labeling on a Green Fluorescent Protein Through Lys–His Linchpin-Directed Modification

**DOI:** 10.3390/s26134095

**Published:** 2026-06-27

**Authors:** Stefania Bova, Marialaura Marchetti, Ilaria De Nardis, Serena Faggiano, Samanta Raboni, Alessandra Gritti, Elisa Pianta, Valentina Pirovano, Giorgio Abbiati, Gloria Modafferi, Barbara Pioselli, Stefano Bruno, Barbara Campanini, Stefano Bettati, Luca Ronda

**Affiliations:** 1 Biopharmanet_TEC, University of Parma, Parco Area delle Scienze, 27/A, 43124 Parma, Italy; stefania.bova@unipr.it (S.B.); ilaria.denardis@unipr.it (I.D.N.); stefano.bruno@unipr.it (S.B.); barbara.campanini@unipr.it (B.C.); 2 Department of Medicine and Surgery, University of Parma, Via Volturno, 39, 43125 Parma, Italy; marialaura.marchetti@unipr.it; 3 Department of Food and Drug, University of Parma, Parco Area delle Scienze, 27/A, 43124 Parma, Italy; serena.faggiano@unipr.it (S.F.); samanta.raboni@unipr.it (S.R.); 4 Institute of Biophysics, National Research Council, Via Giuseppe Moruzzi, 1, 56124 Pisa, Italy; 5 Department of Pharmaceutical Sciences, General and Organic Chemistry Section “A. Marchesini”, University of Milan, Via Golgi 19, 20133 Milano, Italy; alessandra.gritti@unimi.it (A.G.); elisa.pianta@unimi.it (E.P.); valentina.pirovano@unimi.it (V.P.); giorgio.abbiati@unimi.it (G.A.); 6 Department of Chemistry, University of Milan, Via Golgi 19, 20133 Milano, Italy; 7 Chiesi Farmaceutici S.p.A., Global Research and Preclinical Development Area, Largo Belloli, 11/A, 43122 Parma, Italy; g.modafferi@chiesi.com (G.M.); b.pioselli@chiesi.com (B.P.)

**Keywords:** protein biosensors, green fluorescent protein (GFP), site-selective labeling, linchpin-directed modification (LDM)

## Abstract

**Highlights:**

**What are the main findings?**

- Lys–His linchpin-directed modification selectively labels GFP while preserving fluorescence and pH responsiveness.
- LDM chemistry can access cavity-located residues that are not typically targeted by conventional surface labeling.

**What is the implication of the main findings?**

- Protein structure and local microenvironment may determine the modification site beyond geometric matching alone.

**Abstract:**

Protein-based biosensors require controlled and site-selective functionalization strategies to enable stable and oriented immobilization without compromising protein structure and signal transduction efficiency. We evaluated a chemoselective linchpin-directed modification (LDM) approach targeting Lys–His pairs as a tool for site-specific labeling of the model fluorescent biosensor green fluorescent protein (GFP). LDM molecules with variable spacer lengths were prepared, and a structure-guided computational workflow was implemented to map Lys–His distances on the protein and identify candidate modification sites. Experimental validation by UV-Vis spectroscopy and mass spectrometry demonstrated efficient conjugation and a final degree of labeling close to unity, consistent with single-site modification, with all LDM molecules selectively targeting the same histidine residue (His181), independently of spacer length. Structural analysis revealed that this residue is located within an accessible internal cavity that favors productive interactions with the reactive group. Importantly, the modification preserves GFP fluorescence and pH response, confirming retention of sensing functionality. These results demonstrate that LDM enables selective modification not only of surface residues, but also of structurally guided, non-surface residues. This approach provides the proof of concept of a new, promising strategy for the controlled functionalization and immobilization of protein-based biosensors.

## 1. Introduction

**Figure 1:**
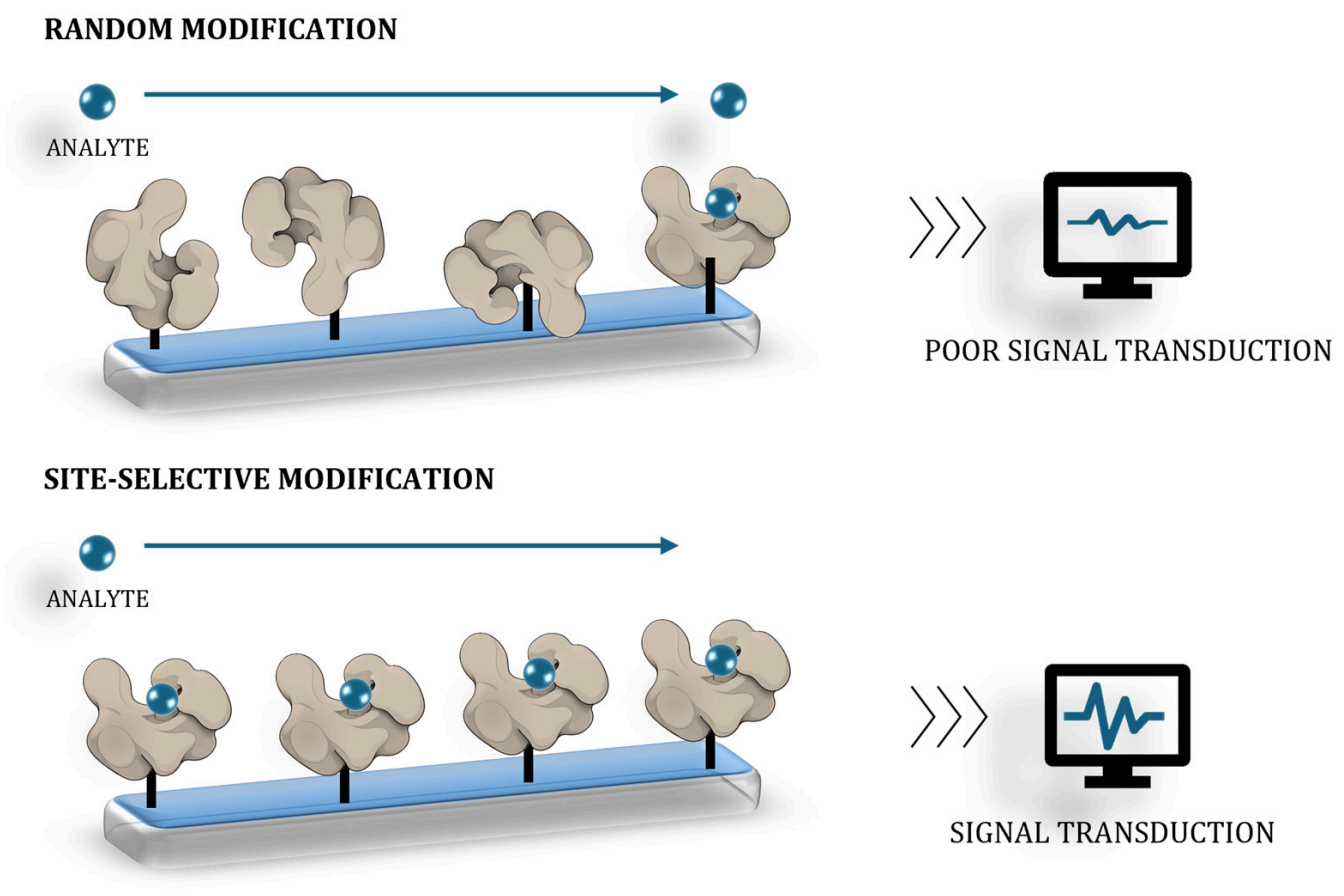
An example of the advantage of site-selective chemical modification for the immobilization of a protein-based sensor vs. non-selective random modification.

Protein-based biosensors include both genetically encoded sensors, which are expressed in living cells to monitor analytes and signaling events [1,2,3] and in vitro systems, based on purified proteins chemically modified to transduce sensing event [4,5,6,7]. In the latter class, biosensor performance is tightly linked to preservation of the native conformation and activity of the recognition element during chemical functionalization and surface coupling. Therefore, identifying protein sites that can be modified without perturbing function is a necessary preliminary step in the development of robust protein-based biosensors. As a further source of complexity, amino acids targeted by conventional chemical modifications are typically present at multiple sites, and distinct protein populations with significant variability in functionality and stability can be generated (Figure 1). To achieve site-selective modifications, the protein should contain a uniquely reactive functional group, such as, for example, a sulfhydryl group on a single cysteine side chain. However, it is common that proteins have no cysteines or have more than one; for this reason, site-directed mutagenesis is often necessary to substitute multiple reactive residues with unreactive ones, further complicating the procedure.

**Figure 2:**
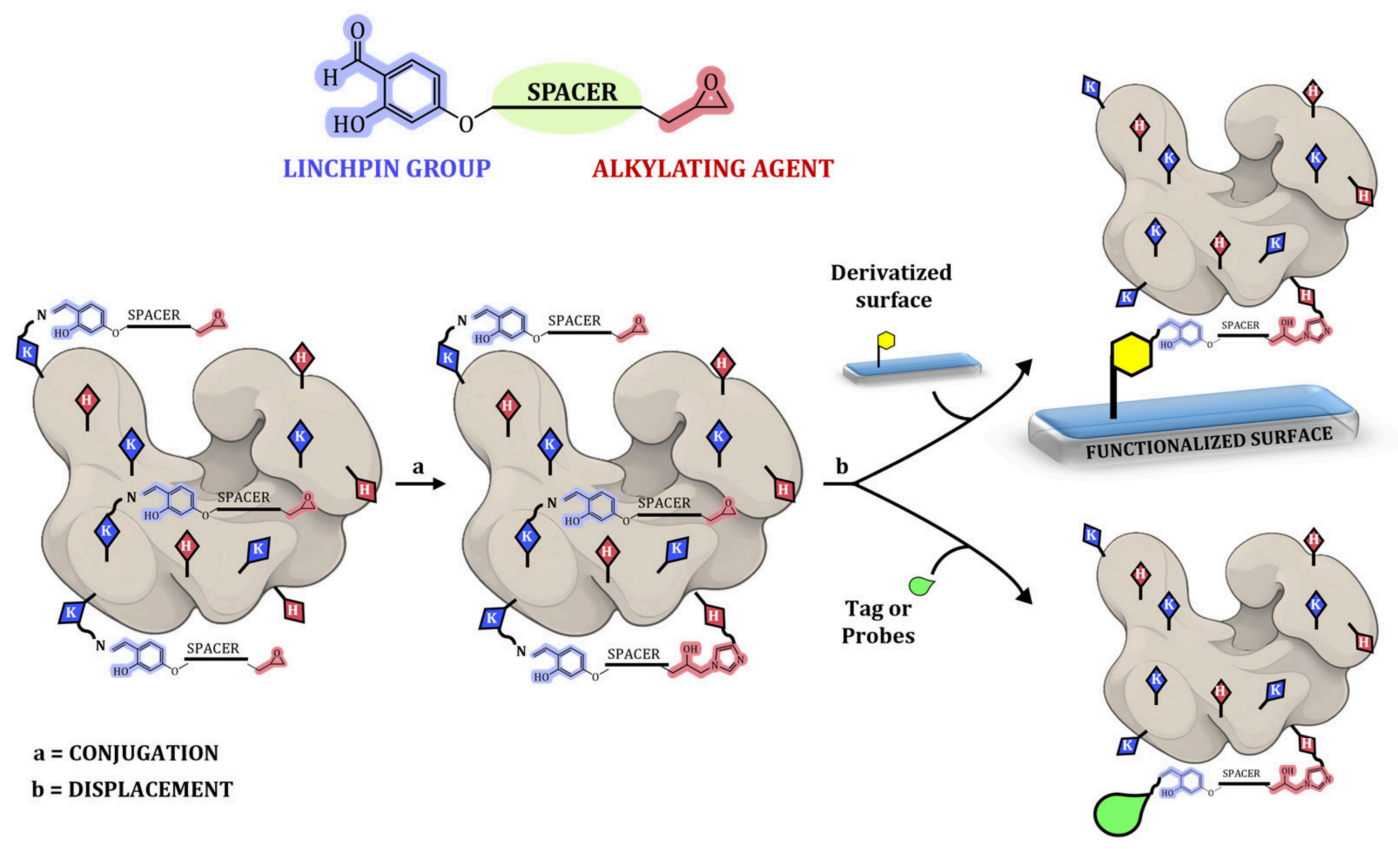
A general scheme of the mechanism of reaction of LDM molecules towards lysine and histidine pairs.

An intriguing alternative that combines regio- and chemoselectivity is represented by the linchpin-directed modification (LDM) platform, which exploits bifunctional molecules with two electrophilic centers joined by a spacer of variable length [8] (Figure 2). One reactive group, named the linchpin group, produces a reversible, chemoselective reaction with residues typically present in several copies on the surface of proteins (e.g., lysines), while the second one alkylates with a slower reactivity another amino acidic residue that falls within the reaching distance of the chosen linker. The latter covalent handle, considering the reversibility of the first reaction, offers a further opportunity to bind spectroscopic probes or tags, or to act as an anchoring point for surface functionalization reactions [9]. For example, the use of LDM to achieve ordered LDM molecules targeting amino acid pairs [10,11,12] has been described.

Building on these reports, the present study focuses on Lys–His pairs, whose high abundance on protein surfaces enables to cover a wide range of proteins and the selection of an optimal modification site. The linchpin moiety is represented by 2-hydroxybenzaldehyde, which enables the formation of a stable Schiff base with lysine side chains under mild reaction conditions, while retaining reversibility. The hydroxyl substituent in ortho position further contributes to stabilizing the carbonyl functionality against oxidation under physiological conditions throughout the reaction. In contrast, the alkylating agent consists of an epoxide group, selected for its high chemoselectivity toward histidine residues. The general molecular architecture and mechanism of LDM molecules have already been described [8]. A concise overview of the approach is reported in Figure 2.

To explore the potential of the LDM approach to develop protein-based biosensors, we focused on: (i) the design and synthesis of a series of LDM molecules for Lys–His pairs with spacers of variable length, exhibiting solubility and reactivity compatible with low temperature and protein-friendly solvent conditions (low fraction of apolar solvent); (ii) the optimization of reaction protocols through absorption spectroscopy; and (iii) the testing of LDM on a model sensor protein, green fluorescent protein (GFP). In particular, we sought to determine whether, beyond enabling access to distinct single sites on proteins, structural information could be used to predict and interpret the final site of modification obtained with LDM molecules. Specifically, we investigated whether a structure-based analysis of the protein reactivity landscape could guide the selective modification of a target amino acid side chain by modulating the linker length, i.e., the relative positioning of the two reactive centers within LDM molecules.

A GFP variant was selected as an archetypal protein sensor due to several advantageous features: (i) its rigid structure, which guarantees that inter-residue distances derived from crystal structures closely approximate those in the solution; (ii) its high stability, which allows to explore a wider range of reaction conditions, while ultimately aiming to avoid harsh temperatures, extreme pH values, and non-compatible solvents; and (iii) the presence of an intrinsic fluorophore, which underlies its sensing function and provides a convenient readout for monitoring protein integrity and stability. Specifically, we used the well-characterized GFP triple mutant Ser65Ala, Val68Leu, Ser72Ala, called GFPmut2, endowed with highly efficient folding at 37 °C, enhanced fluorescence emission, and increased pK_a_ of the chromophore [13,14]. Lys–His couples were scanned in silico over the whole sequence, and their relative distances were mapped based on the crystallographic structure of the protein and compared to corresponding distances between the two reactive centers on the synthesized LDM molecules. This matching was used to assess whether the site of modification could be predicted and to explore the possibility of creating a LDM toolkit for directing chemical modification on a specific amino acid without the need for extensive protein mutagenesis.

The tested molecules were able to attach to GFP demonstrating their capacity of directing on a specific site. Mass spectrometry (MS) analysis revealed that the site of labeling, while compatible with structural computational evaluation, is the same for all the molecules, suggesting that the spacer did not show to be relevant in the selection of the conjugation site. Indeed, structural evaluation of the modified histidine found it as a solvated, accessible residue, even though not fully exposed on the protein surface. This unexpected feature resulted advantageous, likely allowing LDM positioning in a funnel and thus increasing the local concentration and the reaction rate, with respect to the freely exposed histidines.

These findings suggest that LDM reagents can operate beyond conventional surface labeling, enabling access to buried residues located within cavities through a mechanism in which protein structure, rather than linker design alone, determines the final site of modification. If this class of sites is present on the protein, they may represent attractive targets for protein-based biosensors as they could offer anchoring points less susceptible to degradation.

## 2. Materials and Methods

### 2.1. Reagents

Reagents, if not otherwise specified, were obtained from Sigma Aldrich (St. Louis, MO, USA) at the best commercially available quality. Unless otherwise specified, all experimental tests were carried out in 50 mM Na phosphate buffer at pH 8.0. Spectroscopic measurements were performed using a Cary 4000 UV–Vis spectrophotometer (Agilent Technologies, Inc., Santa Clara, CA, USA), equipped with a thermostatic water bath. Data analyses and graphical representations were performed using MATLAB^®^ R2022b (MathWorks, Natick, MA, USA) and Sigmaplot 12.5 (Systat Software Inc., Chicago, IL, USA) software.

### 2.2. LDM Molecules

**Scheme 1:**
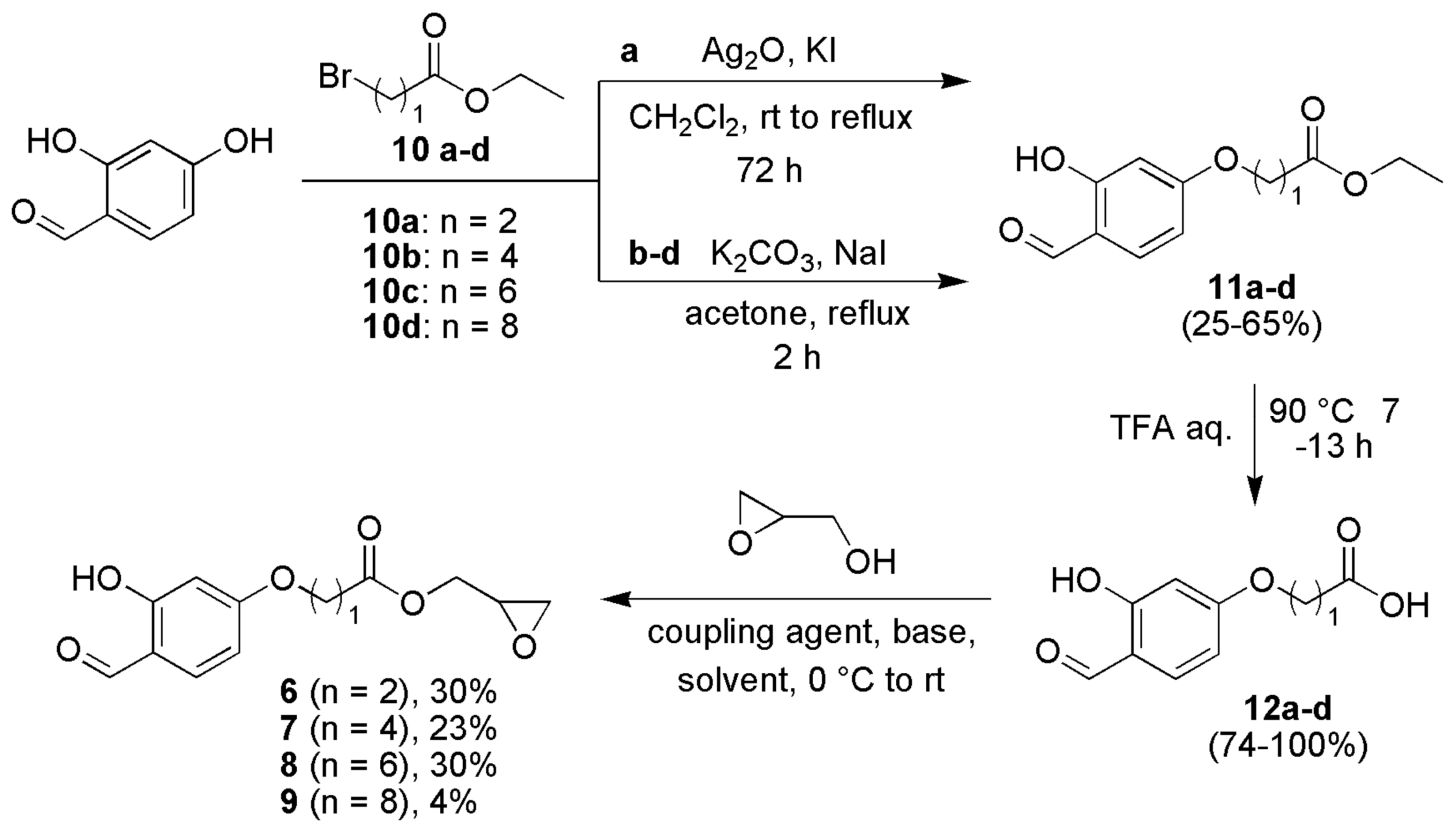
Synthetic approach to LDM molecules **6**–**9**.

LDM molecules **1–4** were purchased from standard commercial suppliers. LDM molecule **5** was prepared following the procedure previously reported by Rai and co-workers [8]. New LDM molecules (**6–9**) were synthesized following the approach described in Scheme 1 (see Supplementary Materials for experimental details and evidence).

Commercially available 2,4-dihydroxybenzaldehyde was reacted with four different ω-bromo-ethyl esters (**10a-d**), each bearing alkyl chains of varying lengths (3, 5, 7, and 9 carbons, respectively). The reactions were carried out using potassium carbonate and sodium iodide, as the base and promoter, respectively, in refluxing acetone as the solvent, affording the corresponding ethers **11b-d**, in yields ranging from 45 to 65%. However, these conditions were ineffective for the synthesis of **11a**. To overcome this issue, silver(I) oxide and potassium iodide in dichloromethane were used to promote halide activation and facilitate the nucleophilic substitution reaction, which successfully yielded the desired product **11a** in 26% yield.

Then, the ester groups of compounds **11a-d** were hydrolyzed to the corresponding carboxylic acids by treatment with aqueous trifluoroacetic acid at 90 °C, achieving the desired products **12a-d** in excellent yields. Finally, the desired LDMs **6–9** were obtained by a coupling reaction with glycidol in the presence of different dehydrating systems (coupling agent + base), in particular, dicyclohexylcarbodiimide/4-dimethylaminopyridine (DCC/DMAP) for LDMs **6** and **7**, 1-ethyl-3-(3-dimethylaminopropyl)carbodiimide/DMAP (EDC/DMAP) for **8** and EDC/N,N-diisopropylethylamine/DMAP (EDC/DIEA/DMAP) for **9**. The desired LDMs **6**–**9** were obtained in yields ranging from 4 to 44% (see Supporting Information for experimental details).

All LDM molecules, summarized in Table 1, were dissolved in 100% dimethyl sulfoxide (DMSO) to obtain 12 mM stock solutions for conjugation protocols. Stock solutions were stored at −80 °C.

### 2.3. Computational Measurements on LDM Molecules

Structure-based predictions of LDM molecule chemical properties were performed using Chemicalize (Chemaxon, Budapest, Hungary). The spacer distances of LDM molecules, defined as the maximum distance between the aldehyde carbonyl group of the linchpin moiety and the epoxide carbon of the alkylating agent targeted by the nucleophilic attack of the histidine Nε2 imidazole nitrogen, was measured using Discovery Studio Visualizer 2021 (BIOVIA, Dassault Systèmes, Paris, France), considering molecules arranged in the more extended conformation. Solvent-accessible surface area (SASA) was calculated using PyMOL Molecular Graphics System, Version 3.0 (Schrödinger, LLC, New York, NY, USA).

### 2.4. Physico-Chemical Characterization of LDM Molecules

The solubility of LDM molecules was evaluated through spectroscopic techniques. After solubilization, the molecules were diluted to a final concentration of 100 μM in solutions containing 10%, 5%, or 1% DMSO (*v*/*v*), and an initial absorption spectrum was recorded immediately (time zero). The samples were subsequently centrifuged at 16,000× *g* for 10 min at room temperature, after which a second absorption spectrum was recorded. The dependence of absorption as a function of compound concentration was evaluated by collecting absorption spectra at different compound concentrations (10, 30, 50, and 100 μM).

Fluorescence emission spectra of the compounds were recorded using an FS5 spectrofluorometer (Edinburgh Instruments Ltd., Livingston, Great Britain) with slits set at 5 nm, and quartz cuvettes with 0.3 cm optical path length. LDM molecules were prepared at 5 μM concentration in Na phosphate buffer, with DMSO concentration reduced to 1%, and excited at their absorption maxima.

### 2.5. Characterization of LDM Molecules Reactivity Toward Nα-Acetyl-L-Lysine

The reaction between the linchpin group of LDM molecules with amino groups was monitored by UV-Vis spectroscopy, using Nα-acetyl-L-lysine (NAL) as a model compound. LDM molecules at 500 μM concentration were combined with NAL at a 1:4 molar ratio, corresponding to a final concentration of 2 mM. The reaction (shown in Scheme S1) was monitored in a 0.1 cm optical pathlength cuvette, over approximately 24 h, by collecting absorption spectra at specified time intervals: every 15 min during the first two hours, followed by hourly measurements.

### 2.6. Computational Characterization of GFP Structure and Reactivity

Structural analyses were carried out on the GFP structure (PDB: 1GFL), using a combination of computational tools. Firstly, intermolecular contact distances between lysine and histidine residues were accurately estimated based on the atomic distances obtained in the PDB structure, using the NCONT module implemented in Collaborative Computational Project Number 4 (CCP4) software suite, version 7.1 [15]. Measurements were taken between the nitrogen of the amine group of the lysine side chain and the nitrogen Nε2 of the imidazole ring of histidine. The spatial orientation of residues falling within distance ranges compatible with the LDM spacer length was further examined using UCSF Chimera 1.15 [16] to identify the most probable rotamers. Finally, lysine and histidine reactivity was mapped using the computational web tool propKa, version 2.0 [17].

### 2.7. Protein Expression and Purification

The recombinant protein was overexpressed in *E. coli* TUNER™ BL21(DE3) cells transformed with a pET28b(+) expression vector, carrying the gene coding for His-tagged GFPmut2 [13] gene, in Luria-Bertani (LB) broth. After induction, performed for 3 h at 37 °C in the presence of 1 mM isopropyl-β-D-thiogalactopyranoside (IPTG), cells were harvested by centrifugation and washed once with phosphate buffered saline (PBS). The cell pellet was resuspended in lysis buffer consisting of 50 mM Na phosphate buffer pH 8.0, 300 mM NaCl, added of 1 mg/mL lysozyme, 0.2 mM phenylmethylsulfonyl fluoride (PMSF), 0.2 mM benzamidine and 1.5 μM pepstatin A. After 45 min incubation under agitation, cells lysis was conducted by sonication, and the suspension was centrifugated to separate the soluble fraction from the debris. The recombinant protein was purified in batch using 3 mL of pre-equilibrated Talon metal affinity resin (Cytiva, Wilmington, DE, USA). After flow-through elution, the resin was washed with a lysis buffer enriched with 1% glycerol, and the protein was eluted using a buffer containing 500 mM imidazole. The solution was diafiltrated, reducing the imidazole concentration below 1 mM and dialysed in PBS, in the presence of thrombin (15 U/mg of protein) to promote His-tag cutting. After proteolytic treatment, sample purity was assessed using SDS-PAGE analysis. Protein concentration was calculated by using an extinction coefficient of the chromophoric group at 485 nm of 57,800 M^−1^ cm^−1^ and a molecular weight (MW) of 27,416 g/mol. Protein aliquots were flash-frozen in liquid nitrogen and stored at −80 °C until further use.

### 2.8. LDM Conjugation Protocol and UV-Vis Spectroscopy Characterization

**Scheme 2:**
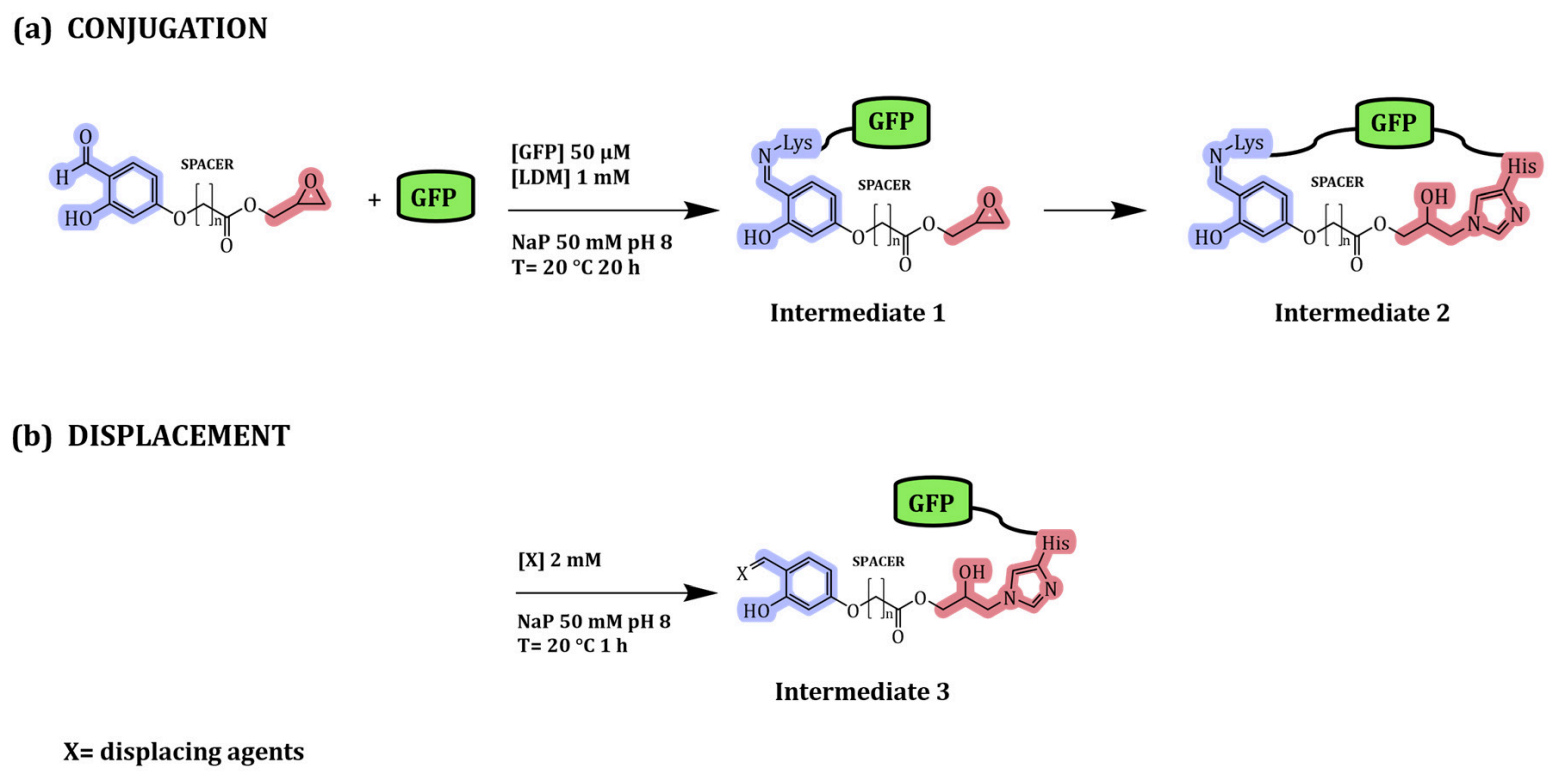
GFP conjugation with LMD molecules and displacement step. The displacing agents X used were hydroxylamine, hydrazine, or phenylhydrazine.

The spectroscopic properties of LDM molecules were exploited to study the stages of the multistep modification mechanism ([8] and Figure 2). GFP conjugation with LDM molecules, illustrated in Scheme 2, was performed by incubating the protein with LDM at 1:20 ratio, corresponding to a final concentration of 50 μM and 1 mM LDM molecules, while maintaining 1% (*v*/*v*) of DMSO. The reaction was monitored for 20 h following the variation of LDM molecules absorption signal, by collecting spectra every 30 min during the first two hours, followed by one measurement every hour, using a 0.1 cm path-length cuvette and maintaining the temperature at 20 °C.

Following LDM conjugation, hydroxylamine, hydrazine, or phenylhydrazine was incubated at 2 mM for 1 h at 20 °C (displacement in Scheme 2) to displace the reversible bond by reaction with the imine group of intermediate 1 or 2. After treatment with the displacing agents, unreacted reagents were removed by diafiltration using Amicon^®^ Ultra centrifugal filters with a 10 kDa MW cut-off (Merck-Millipore, Darmstad, Germany). Absorption spectra of the final products were recorded using a 0.3 cm path length cuvette and 70 μL of undiluted samples to estimate protein and probe concentrations, based on their respective extinction coefficients (Table S1). DOL (degree of labeling) for each reaction step and for all the tested LDM molecules was calculated from the ratio between probe and protein concentrations. For the conjugation step, a reducing agent, sodium cyanoborohydride, was added at 2 mM concentration for 1 h at 20 °C, to stabilize the conjugates and enable accurate estimation of the DOL. Absorption spectra of purified samples were recorded under the same experimental conditions described above.

Absorption spectra of modified GFPmut2 at different pH were collected in a solution containing 100 mM NaH_2_PO_4_/Na_2_HPO_4_, 10 mM sodium citrate at different pH at 25 °C, and absorbance at 485 nm was plotted against pH and fitted to Equation (1) [18]:

(1) $$F=a+\left(b-a\right)\frac{10^{n\left(pK_{a}-pH\right)}}{1+10^{n\left(pK_{a}-pH\right)}}$$

where *F* is the fluorescence intensity, *a* and *b* are the fluorescence intensities at acidic and alkaline pH values and *n* is an empirical Hill coefficient.

### 2.9. HPLC-MS Analyses

GFP samples (reacted with molecule **4** and displaced with hydroxylamine) intended for intact protein analysis were analyzed using LC–MS through direct injection without prior treatment. Only molecule **4** was used for this measurement to maximize the mass increase upon derivatization. Chromatographic separation was carried out on a SecurityGuard C18 cartridge (4 × 2 mm) coupled to an LTQ Orbitrap XL mass spectrometer (Thermo Fisher Scientific, Waltham, MA, USA). Elution was carried out using a mixture consisting of eluent A (water containing 0.1% formic acid) and eluent B (acetonitrile containing 0.1% formic acid). A constant flow rate of 0.2 mL/min was applied by setting the following gradients: 0–2 min isocratic to 1% eluent B; 2–3.5 min linear gradient from 1 to 80% eluent B; 3.5–6 min isocratic to 80% eluent B; 6.0–6.5 min gradient from 80% to 1% eluent B; 6.5–10 min reconditioning. Injections (5 μL) were performed after three-fold dilution in eluent A.

Samples designated for peptide level analysis (reacted with molecules **5**, **6** and **7**) were first subjected to chromatographic separation and subsequently to enzymatic digestion prior to LC–MS analysis. Chromatographic separation was performed using a Shimadzu Prominence LC-20A HPCL system (equipped with CMB20 communications module, DGU-20A degassing unit and SPD-20A UV-Vis detector; Kyoto, Japan) and a reversed-phase Avantor^®^ ACE^®^ C4, HPLC Columns, 5 μm particle size, 250 mm length and 4 mm diameter (Radnor Township, PA, USA), maintaining the temperature of 45 °C using a GECKO-2000^®^ HPLC Column Heater G3080 (Amchro, Hattersheim, Germany). For each sample, 20 μL of 2 mg/mL protein solution, corresponding to 40 μg, was injected maintaining a constant flow rate of 0.4 mL/min. The following gradient was used, generated by mixing eluent A (water + 0.1% formic acid) and eluent B (acetonitrile with 0.1% formic acid): 0 min 90% eluent A; 5 min 90% eluent A; 25 min 50% eluent A; 30 min 50% eluent A; 35 min 10% eluent A; 40–45 min reconditioning. Each eluted chromatographic fraction was dried using a SpeedVac concentrator RVC 2-18 (Martin Christ Gefriertrocknungsanlagen GmbH, Osterode am Harz, Germany) and resuspended in 50 mM ammonium bicarbonate buffer, pH 8.0, to approximately 1 mg/mL protein concentration.

Digestion was performed after an initial reduction and alkylation step, followed by cleavage with chymotrypsin. The samples were reduced with 6 mM dithiothreitol (DTT) and incubated at 95 °C for 5 min, then alkylated with 10 mM iodoacetamide for 30 min at room temperature in the dark. The reaction was quenched by adding DTT at the same concentration, reacting for 15 min at room temperature. Due to the high stability of GFP to protease activity, chymotrypsin digestion was preceded by a mild protein denaturation step from the addition of a denaturing agent (0.1% SDS) and heating at 95 °C for 20 min. Calcium chloride (2 mM), required for chymotrypsin activity, was added to the digestion buffer, and then samples were incubated at 37 °C for 6 h with chymotrypsin (1:10 molar ratio with respect to the protein). Enzymatic cleavage was stopped by the addition of trifluoroacetic acid (TFA) to a final concentration of 0.1% (*v*/*v*). To determine the modification site, GFP and GFP derivatized with molecules **5**, **6**, and **7** were reduced, alkylated, digested with chymotrypsin, and desalted with desalting spin columns (Thermo Fisher Scientific, Waltham, MA, USA). The digested desalted peptides were dried under vacuum and resuspended in 30 µL of 0.1% trifluoroacetic acid in water. An aliquot of 2 µL of peptides was separated by a Dionex UltiMate3000 nanoUHPLC system equipped with a PepMap RSLC C18 column (Thermo Fisher Scientific) at 35 °C using a four step gradient at a flow of 0.3 µL/min (80% acetonitrile in 0.1% formic acid from 1% to 28% in 100 min; from 28% to 45% in 20 min; from 45% to 90% in 5 min; 14 min at 90% and 36 min of column equilibration at 1%). Peptide fractions were analyzed using an Orbitrap Exploris 240 mass spectrometer (Thermo Fisher Scientific, Waltham, MA, USA) operating in data-dependent acquisition (DDA) mode, with the most abundant ions selected for fragmentation by collision-induced dissociation (CID). Peptide mapping and data analysis were performed using BioPharma Finder Software 2.5 (Thermo Fisher Scientific, Waltham, MA, USA) setting the protein sequence of GFP as a reference and the GFP-LDM conjugates listed in Scheme 2 as static modifications (intermediate 1 on lysine residue, intermediate 2 on lysine and histidine residues, intermediate 3 on histidine residue).

## 3. Results

### 3.1. Design and Synthesis of LDM Molecules

We generated a panel of LDM molecules starting from a previously published series endowed with spacers of different lengths and chemical nature [8], and that was already shown to react with lysine and histidine pairs (Table 1, molecules **1–5**). To extend the range of Lys–His distances that can be covered by the linker, new compounds based on molecule **5** were designed and synthesized. Molecules **6** and **7** differ from molecule **5** only for the removal and addition of one methylene group, respectively. These molecules were used to investigate the effect of the length of the spacer on directing the reaction to different sites but also to determine if a methylene group can be considered the minimal unit sufficient to differently direct the conjugation. Then, with molecules **8** and **9**, we further increased the spacer length to widen possible coupling distances (Table 1).

**Table 1:** LDM molecule structure, MW and calculated distance between the aldehydic carbon of the linchpin moiety and the epoxide carbon of the alkylating moiety (considering molecules arranged in the more extended conformation).

Molecule	Structure	MW (g/mol)	Distance (Å)
**1**	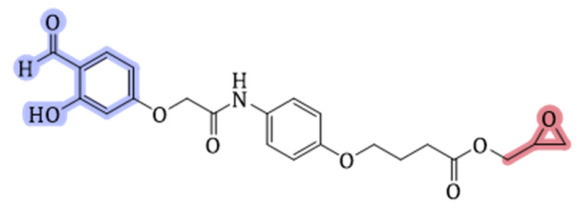	429.4	17.4
**2**	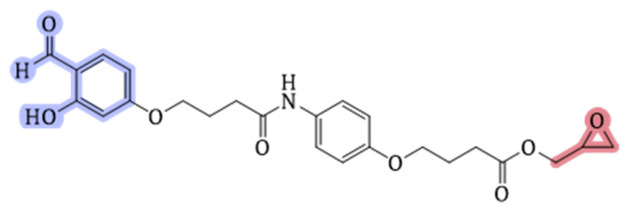	457.5	18.1
**3**	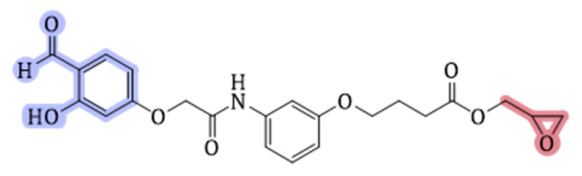	429.4	18.2
**4**	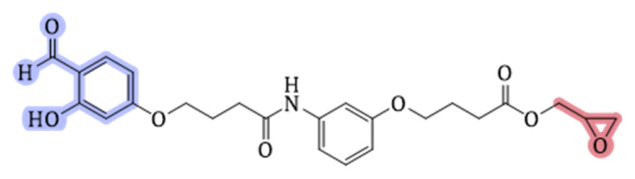	457.5	23.0
**5**	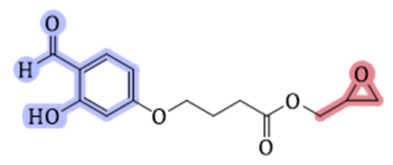	280.3	13.6
**6**	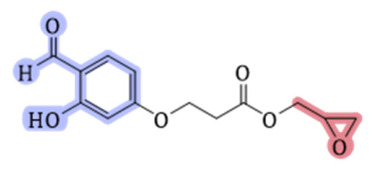	266.3	12.5
**7**	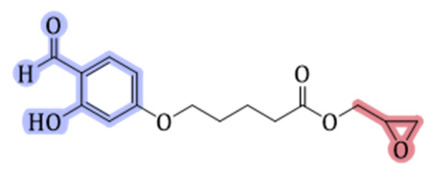	294.3	15.1
**8**	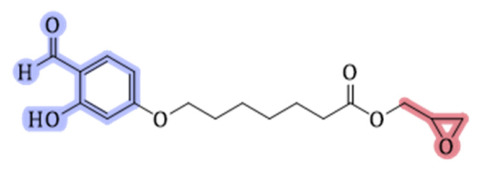	322.3	18.8
**9**	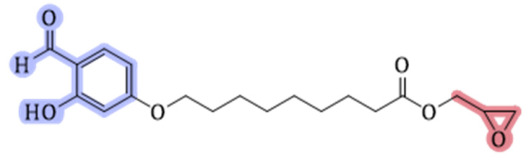	350.0	21.5

### 3.2. Structural and Physicochemical Characterization of LDM Molecules

A computational structural analysis of the maximum distance between the reactive centers was carried out for each molecule to define the available reaction space once the first moiety was attached to the protein (Table 1). Distances cover a range from 12.5 Å (molecule **6**) to 21.5 Å (molecule **9**), assuring a wide range of contact possibilities with respect to a typical protein surface (Table 1).

In addition, key molecular properties, such as solubility in water, were estimated through the dedicated software Chemicalize (Table 2). The theoretical solubility in water highlighted that the aromatic ring in the spacer, present in molecules **1**–**4**, lowers the solubility of the LDM molecule to less than 0.2 mg/mL. Even removing the aromatic ring, an increase in the number of methylene groups above 4 resulted in a drop of water solubility (molecules **8** and **9**). Solubility was then experimentally evaluated by preparing 1 mM stock solution of the molecules in water added of 1% DMSO and exploiting the spectroscopical features of the molecules, since the benzaldehyde group exhibits absorption maxima at 363 and 281 nm and fluorescence emission at 490 nm (Figure S1). These signals provide a straightforward method to determine the concentration and solubility of the LDM molecules (Figure S2).

Experimental data confirmed the solubility trend, since only molecules with predicted solubility equal to or greater than 0.5 mg/mL were shown to be experimentally soluble as 1 mM stock solution with 1% DMSO (Table 2). Also, the pK_a_ values were computationally estimated, resulting to be 8.91 for all molecules.

**Figure 3:**
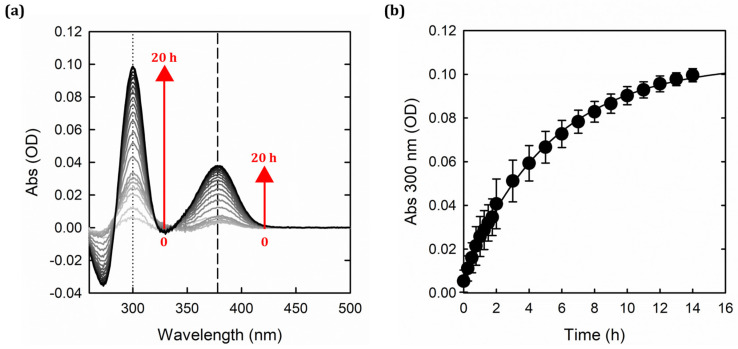
Spectroscopic measurements after reaction of LDM molecule **5** with NAL. (**a**) Difference spectra as a function of time. Spectra were collected every 15 min for 2 h and then every hour for 14 h. The grayscale gradient indicates spectral time evolution, from light gray to black. (**b**) Reaction kinetic followed through absorbance at 300 nm.

To mimic the reactivity of amine side chains in a protein, the LDM molecules were reacted with NAL, an amino acid derivative in which only the side chain Nε is available for the reaction with an aldehyde group, thus mimicking the reaction with a lysine residue within a protein sequence (Scheme S1). The reaction of the LDM molecules with NAL caused spectral changes: difference spectra with respect to that of the unreacted molecules showed variations in the UV-Vis range, with maximum differences at 300 and 378 nm (spectra for molecule **5** are reported as an example in Figure 3a). The time course of the absorbance signal at 300 nm shown in Figure 3b was fitted to an exponential equation, yielding a half-life time of 5.44 ± 0.18 h. This suggests that the reaction can take place in a time frame typical of protein sensor functionalization protocols.

### 3.3. Structure-Based Analysis of GFP Reaction with the LDM Molecules

After characterizing the LDM molecules and their reactivity towards the target amino acid under protein-compatible conditions, we proceeded to evaluate the application of this chemistry on a more complex system, selecting GFP as a model sensor protein. GFP and its engineered variants are widely employed as fluorescent biosensors, with a broad range of GFP variants developed to modulate spectral properties and confer sensitivity to environmental parameters such as pH, redox state, and ligand binding [19]. In this context, we considered the possibility to introduce covalent modifications at defined positions on GFP variants with different sensing capabilities as a valuable objective.

A necessary condition for the reaction to occur is that the distance between the two centers on the LDM molecules is compatible in terms of length and accessibility to allow both reactions on the protein. The nature of the spacer is determinant in this chemical modification approach and is proposed to reach different, single sites on the protein.

In view of chemically modifying a protein sensor for labelling or its anchoring on a solid support or surface, detailed structural information on the protein is required to identify which surface regions or residues are more suited to sustain a chemical modification without altering the protein sensing function. To challenge the potential of LDM approach in these terms, we evaluated if it was possible to predict and direct, through a structure-based analysis of the protein reactivity landscape, the chemical modification on a desired amino acid side chain by modulating the linker length. The proposed structure-based computational method was designed to offer a comparison between GFP inter-residue distances and the spacer length of LDM molecules.

**Figure 4:**
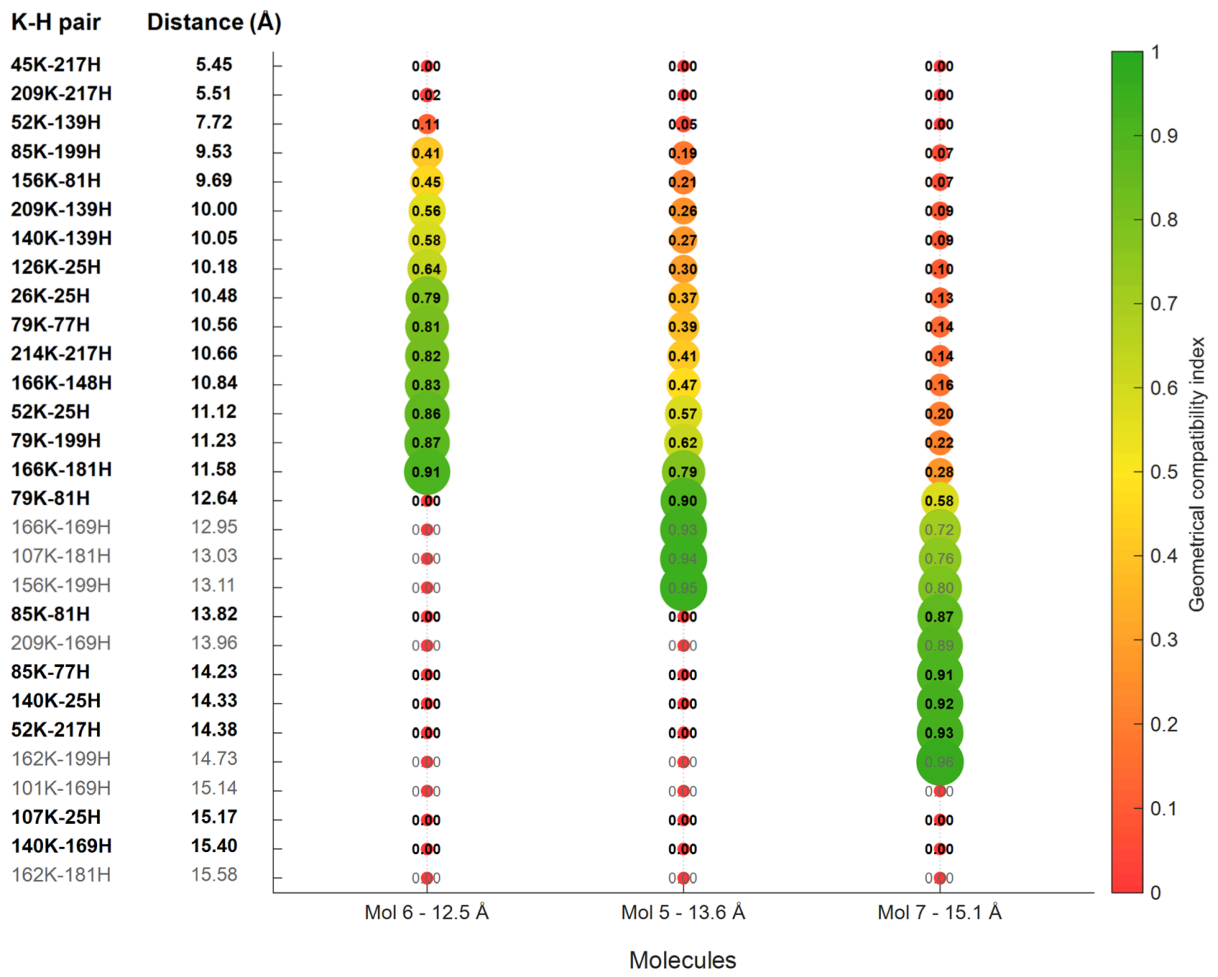
Bubble plot summarizing the geometrical compatibility index for each linchpin–Lys–His pair combination. Numerical values inside each cell report the calculated geometrical compatibility index. Pairs considered geometrically compatible but excluded from interpretation because of the steric clashes (Figure S3) are shown in gray, indicating steric infeasibility despite nominal distance matching.

The NCONT module of CCP4 suite enabled the calculation of all distances between selected histidines and lysines of the protein in a single procedure. We focused our comparison on molecules **5**, **6**, and **7**, in view of following experimental validation, for two reasons: (a) these molecules are the most soluble, with comparable solubility limits, thus avoiding any effect of partial solubilization on modification yield; (b) they differ by only a minimal structural variation, i.e., by the removal (molecule **6**) or addition (molecule **7**) of a single methylene unit relative to molecule **5** (Table 1). The resulting data were visualized as a heatmap (Figure S3), which allows direct comparison between inter-residue distances and the spacer lengths of LDM molecules. This graphical representation provided rapid identification of residue pairs whose distances fell within the range accessible to the distances of the two reactive groups in LDM molecules, while excluding residue pairs located too close or too far relative to the length of the molecules. The final output of this structure-based analysis is a set of residue–distance combinations which were compared with the maximum distance for each LDM molecule. The distance analysis was approached introducing a heuristic geometrical compatibility index, based on the comparison between the Lys–His distances measured in the GFPmut2 structure and the maximum distances among the reactive sites of the different LDM molecules. The index has a maximum value when the inter-residue distance matches the optimized molecular span and progressively decreases for shorter distances. A shallow decay was applied within the first ~2 Å to account for limited conformational flexibility, likely occurring given the rotational freedom of the spacers, followed by a steeper decline beyond this range to penalize increasing molecular distortion, with the score approaching zero at ~7 Å below the optimal distance (Figure S4). Distances exceeding the optimized span were assigned a score of zero, as such geometries are not physically accessible. The matching of distances between reactive groups in LDM molecules and of Lys– His pairs is reported in Figure 4, and the differences between these distances were used to calculate the index (from 1, green, to 0, red).

Although this analysis could provide only a qualitative indication of geometric matching, it emerges that, for each molecule, the geometrical compatibility index pattern is significantly different, consistent with the fact that, if geometric matching was the main determinant of site selection, a single methylene group can be sufficient to differently direct the reaction on different sites, even though not on a unique one. Moreover, structural analysis suggests that several of these residue pairs are unlikely to engage in plausible interactions. Although they are spatially close, they are separated by intervening regions of the protein, preventing any feasible linker-mediated reaction. These pairs (in grey in Figure 4, and in red in Figure S3b) were excluded from further consideration. However, the reactivity of both amino acid side chains cannot be given for granted in all cases, since steric hindrance, local polarity, protonation state, etc., can impact in addition to the geometric distances. An assessment of lysine and histidine intrinsic reactivity, influenced by the surrounding environment, was also included in the computational workflow to evaluate the reactivity landscape. The results returned by propKa web tool, version 2.0 [17] (Table S2) showed that the predicted pK_a_ of lysines ranged from 10.5 (reference standard value) to 9.0. Since the reaction is carried out at pH 8.0, as in most protein chemical-modification protocols, all lysine residues are expected to have only a very small fraction in the unprotonated, nucleophilic form. Consequently, the observed differences should have only a marginal effect on reactivity toward the hydroxybenzaldehyde group, resulting in a slow but kinetically homogeneous multiderivatization process. On the other hand, predicted histidine pK_a_ values showed larger variations. His25, His77, His81, and His217 show pK_a_ values between 6.0 and 7.4 (Table S2). These values are not too far from the canonical pK_a_ of a free histidine (~6.0). In contrast, the remaining residues (His148, His16, and His181) are estimated to have pK_a_ values of 4.0 or lower, suggesting a state in which the imidazole group is completely unprotonated and therefore more prone to react with the epoxide group of LDM molecules.

### 3.4. Experimental Validation of GFP Reactivity with LDM Molecules

GFPmut2 was overexpressed and purified by affinity chromatography in a fast and high-yield procedure (Figure S5). The absorption spectrum of the protein at pH 8.0 shows two peaks, one centered at 278 nm, accounting for the aromatic residues, and one centered at 485 nm attributed to the deprotonated form of the chromophore that has a pK_a_ of about 6.0 [20]. The reactivity of GFP with LDM molecules was then tested. Similarly to the free amino acids, the conjugation of GFP lysine residues can be monitored by exploiting intrinsic spectroscopic properties of LDM molecules. The results would clarify whether adding or removing one methylene in the spacer is sufficient to differently direct the final conjugation on His or whether larger differences or an improved bond rigidity is necessary.

**Figure 5:**
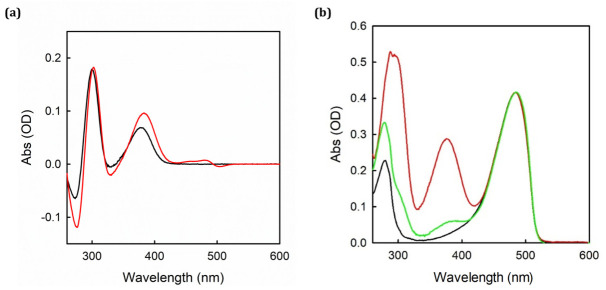
Spectroscopic characterization of LDM molecule **5** reacted with GFP. (**a**) Difference spectrum of LDM molecule **5** with Nα-acetyl-lysine (black curve) superimposes with that of GFP (red curve). Inset: time course of the signal at 302 nm and fitting. (**b**) UV-Vis absorption spectra collected at pH 8.0 on diafiltered product after conjugation and reduction (red curve) and after displacement with hydroxylamine (green curve), compared to the protein before the reaction (black curve). The peak centered at 485 nm corresponds to the GFP chromophore.

Of note, it is not possible to discriminate if the reaction has reached intermediate 1 (benzaldehyde bound to lysines) or has already proceeded to intermediate 2 (Scheme 2), forming a bridge adduct with a histidine residue, because these two intermediates are not expected to be spectroscopically different since the epoxide reaction does not directly perturb the benzaldehyde chromophoric core. Superimposition of the difference spectra with those obtained in presence of NAL confirmed that the observed spectral changes correspond to the conjugation of lysines, as the overall profiles were highly consistent and shared the characteristic band centered at ~300 nm (Figure 5a).

The spectroscopic properties of the LDM molecules allowed real-time tracking of the reaction and estimation of kinetic parameters. The half-life, determined by fitting the absorbance time profile at 302 nm with an exponential equation (Inset Figure 5a), was estimated to be 3.70 ± 0.21 h, a value comparable to that obtained for the free amino acid derivative NAL, which suggests a high and homogeneous reactivity of the lysine residues on the protein. Moreover, the reaction does not seem to perturb the protein structure, as demonstrated by the overlap of the absorption spectrum in the visible region before and after the reaction occurred. The spectroscopic features of LDM molecules were also helpful in determining the DOL, intended as the ratio of probe concentration to total protein fraction.

DOL for molecules **5**, **6**, and **7** was comparable, with an average value of 8.33 ± 0.59, confirming that multi-derivatization occurs, in agreement with the number of available lysine residues on GFP. The following displacement reaction with hydroxylamine brought a decrease in the anchored LDM molecules, in agreement with the mechanism in Scheme 2 and the formation of intermediate 3. For all molecules, the final DOL upon displacement is around 1, supporting a single derivatization for each molecule (Figure 5b).

Since the reaction of epoxide is spectroscopically silent, we confirmed the formation of intermediate 3 (Scheme 2) by MS using molecule **4**. Upon derivatization, the GFP sample was analyzed as an intact protein, and a peak at 27740.77 m/z appeared in addition to the 27276.57 *m*/*z* peak of the unmodified GFP. The mass difference corresponds exactly to the intermediate 3 adduct of molecule **4** with one histidine (464.20 g/mol) and is consistent with the covalent attachment of the molecule to GFP, providing direct evidence that protein conjugation occurs.

The observation that the initial multi-derivatization step led to the formation of intermediate 3 with a DOL of approximately 1 suggests that the reaction proceeds according to the proposed mechanism (Scheme 2). However, at this point, it was not clear if the modified histidine is only one or a distribution of mono-derivatized GFP molecules that occurred and which was (or were) the involved residue(s). This latter information was fundamental to answer our starting question, i.e., if it is possible to predict and direct the covalent modification on a specific amino acid, thus allowing a site-selective labeling and anchoring of a protein biosensor without the need of site-directed mutagenesis.

To determine the modification site, GFPmut2 upon derivatization with molecules **5**, **6**, and **7** and following displacement was digested and MS/MS analysis was performed. The mass spectrometry analysis pointed out that His181 was the only modified residue upon displacement, with no evidence of Lys adduct, as expected from the reaction mechanism (Scheme 2).

**Figure 6:**
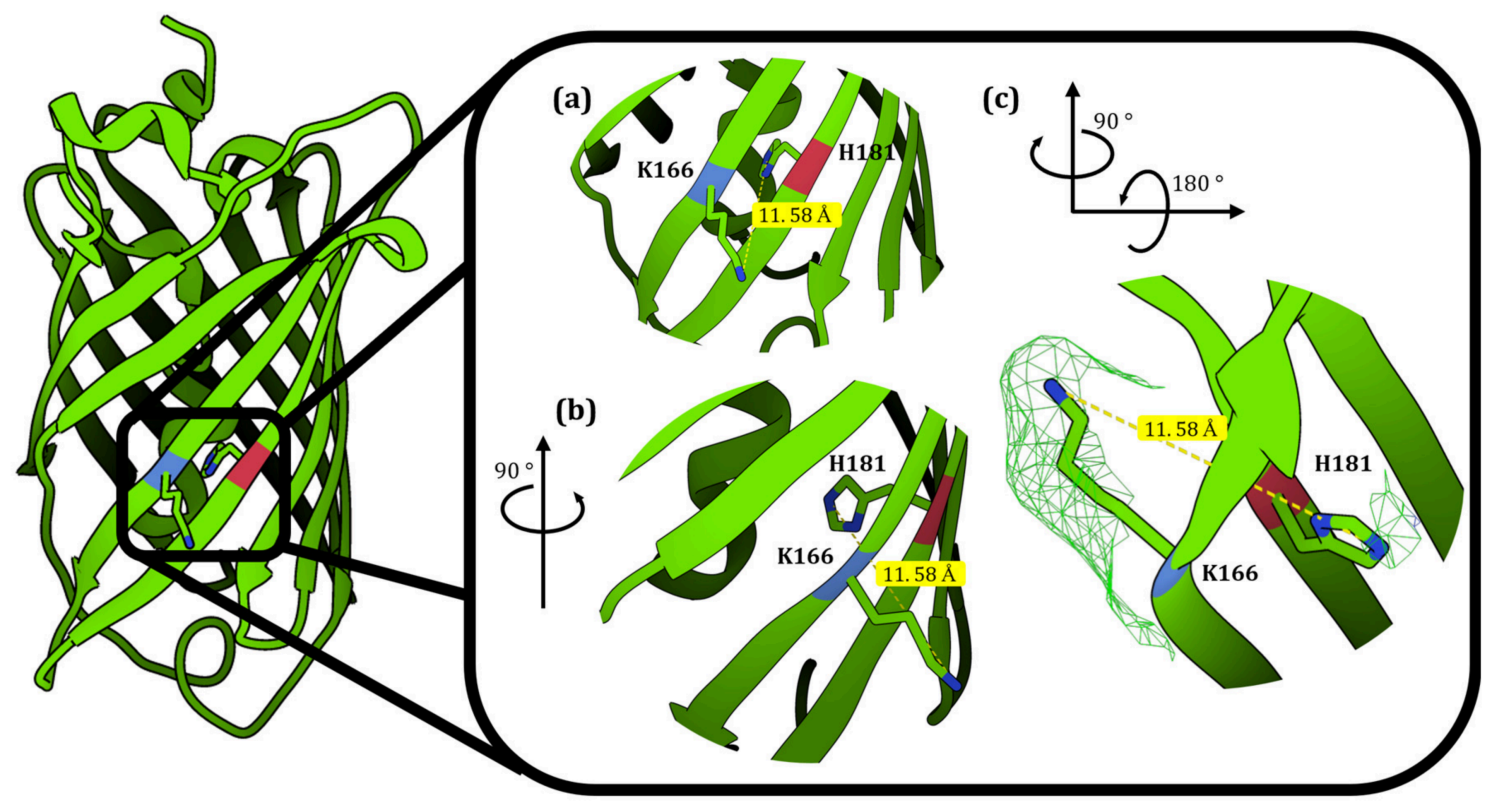
GFP structure with highlighted functionalized His181 and the most plausible coupled lysine during reaction Lys166. The distance is also reported. (**a**) Close-up of the region of interest; (**b**) structure rotated by 90° about the *y*-axis; (**c**) surface mesh after rotation by 90° about the *y*-axis and 180° about the *x*-axis. Lysine and histidine residues are shown in red and blue, respectively.

The experimental results were then structurally evaluated to explain the obtained selectivity. Firstly, we observed that the His181 belongs to the histidine residues with low pK_a_, and hence completely deprotonated at the reaction pH (Table S2). The site of labeling is compatible with structural computational evaluation (geometrical index between 0.28 and 0.91) and between the most reactive ones in terms of pKa. However, this site is the same for all LDM molecules, suggesting that the spacer did not result to be relevant in the selection of the conjugation site. Indeed, structural evaluation of the modified histidine revealed that His181 adopts a conformation in which the imidazole of the side chain in the structure rotated towards the internal part of the barrel, occupying a solvated, accessible position, though not on the protein surface (Figure 6). The calculated solvent-accessible surface area (SASA) for His181 resulted to be 119 Å^2^, confirming that the residue is not deeply buried within the β-barrel. To promote histidine conjugation, the role of Lys166 is hypothesized based on structural analysis. In fact, it is the only available residue based on distance and steric hindrance evaluation (Figure S3).

The LDM reagent is kept in close proximity to the imidazole ring, thereby limiting conformational freedom and increasing the local concentration of the reactive group compared with freely exposed histidines. Within the reaction time window, linchpin anchoring through a lysine residue is therefore expected to accelerate the epoxide reaction with the imidazole, a process that would otherwise require days to weeks in solution [21]. This structural context, together with the reactivity of the imidazole, surrounded by water molecules and available for proton exchange inside the sensor [22], makes this residue particularly suitable for derivatization.

These results highlight an alternative paradigm for biosensors and more generally for protein bioconjugation: usually covalent modification (site selective or not) is achieved exclusively at solvent exposed residues, whereas this chemistry allows modification on internal yet accessible residues. This cavity-directed anchoring does not rely on a specific binding affinity, as observed for ligands targeting enzyme active sites, but rather on reversible interactions with multiple lysine residues on the protein surface, which increase its local concentration, thereby making the subsequent reaction kinetically accessible.

The comparable reactivity observed for LDM molecules endowed with spacers of different lengths indicates that, when this kind of accessible and reactive internal residue is available, spacer architecture is not the primary determinant of site-selectivity. This contrasts with conventional lysine-directed modification strategies, in which spacer geometry often would direct final conjugation on different sites. Instead, we found it possible also that the protein scaffold guides a geometrically constrained funneling effect that directs the reactive group toward a unique, accessible site.

The chemical modification did not perturb the protonation equilibrium of the chromophore; indeed pH-titration of the modified protein allows to estimate a pK_a_ of 5.9 ± 0.4 (Figure S6), in full agreement with data in the literature for the unmodified protein [23]. To our knowledge, His181 is not among the residues that have been involved in the generation of mutants with different spectroscopic features, hence expanding the applicability of this chemistry to the series of sensors based on this protein scaffold.

These findings expand the scope of LDM-type reagents, suggesting that they can operate in both surface-directed and cavity-directed modes, and that their ultimate selectivity can be dictated by protein structure and surface landscape rather than solely by the spacer design. This alternative mechanism offers a strategy to access cryptic, pocket-located functionalization sites that are not addressable through conventional surface-based approaches. We evaluated only GFPmut2, a highly stable β-barrel protein; therefore, the generality of the observed cavity-directed modification mechanism remains to be established on structurally unrelated proteins. Nevertheless, this work suggests the use of this chemistry as a valuable alternative to achieve protein modifications more protected towards degradation reactions that can impair sensor transduction and functioning.

## 4. Conclusions

This work demonstrates that LDM can be applied to GFPmut2 under protein-friendly conditions, yielding selective modification of a single histidine residue while preserving fluorescence and pH responsiveness. Structural and mass spectrometry analyses indicate that all tested LDM molecules converge to the same final modification site and suggest that protein architecture and local microenvironment can play a dominant role in determining the final modification site. Although further validation on additional proteins will be required to assess the generality of this behavior, the observed cavity-directed modification mechanism provides a proof –of concept for accessing cryptic functionalization sites that are not readily addressable through conventional surface-labeling approaches.

## Figures and Tables

**Table 2:** Solubility of LDM molecules: experimental solubility of 1 mM stock solution in water with 1% DMSO and theoretically predicted solubility in water.

Molecules	1 mM Stock (1% DMSO)	Predicted Solubility (mg/mL)
**1**	Insoluble	0.19
**2**	Insoluble	0.15
**3**	Insoluble	0.12
**4**	Insoluble	0.16
**5**	Soluble	4.8
**6**	Soluble	3.7
**7**	Soluble	2.7
**8**	Soluble	0.5
**9**	Insoluble	0.06

## Data Availability

Raw data are available upon reasonable requests to interested researchers.

## Supplementary Materials

The following supporting information can be downloaded at: https://www.mdpi.com/article/10.3390/s26134095/s1, Experimental: LDMs Synthesis and Characterization; Scheme S1: Reaction of the LDM molecules with target free amino acid Nα-acetyl-L-lysine (NAL); Figure S1: Spectroscopic characterization of LDM molecule **5**; Figure S2: LDM molecules solubility characterization; Figure S3: Structure-based selection of GFP residues possibly involved in interaction with LDM molecules; Figure S4: Geometrical compatibility index; Figure S5: SDS-PAGE Tris-Glycine analysis of GFPmut2 purification and UV-vis absorption spectrum of the purified GFPmut2; Figure S6: pH dependence of the absorbance at 485 nm of GFPmut2 modified with molecule **5**; Table S1: Molar extinction coefficients of LDM molecules; Table S2: PropKa analysis. References [8,24,25] are cited in the Supplementary Materials.

## Abbreviations

The following abbreviations are used in this manuscript:

CCP4	Collaborative Computational Project Number 4
DCC/DMAP	Dicyclohexylcarbodiimide/4-dimethylaminopyridine
DMSO	Dimethyl sulphoxide
DOL	Degree of labelling
DTT	Dithiothreitol
EDC/DMAP	1-ethyl-3-(3-dimethylaminopropyl)carbodiimide/4-dimethylaminopyridine
EDC/DIEA/DMAP	1-ethyl-3-(3-dimethylaminopropyl)carbodiimide/N,N-diisopropylethylamine/4-dimethylaminopyridine
GFP	Green fluorescent protein
HPLC	High-performance liquid chromatography
IPTG	Isopropyl-β-D-thiogalactopyranoside
LB	Luria-Bertani
LC–MS	Liquid chromatography-mass spectrometry
LDM	Linchpin-Directed Modification
LTQ	Linear Trap Quadrupole
MS	Mass spectrometry
MW	Molecular weight
NAL	Nα-acetyl-L-lysine
PMSF	Phenylmethylsulfonyl fluoride
SDS-PAGE	Sodium Dodecyl Sulphate-PolyAcrylamide Gel Electrophoresis
TFA	Trifluoroacetic acid
