# Supplementary material for: Exploring the Potential of Site-Selective Labeling on a Green Fluorescent Protein Through Lys–His Linchpin-Directed Modification
Source: Sensors (Basel). 2026 Jun 27;26(13):4095. doi: 10.3390/s26134095 (PMC13364192; doi:10.3390/s26134095)
Supplement: Supplementary file 1 [file sensors-26-04095-s001.zip › sensors-4361368-supplementary.pdf]

Supplementary Materials

Exploring the Potential of Site-Selective Labeling on a Green Fluorescent Protein Through Lys–His Linchpin-Directed Modification

Stefania Bova [1], Marialaura Marchetti [2], Ilaria De Nardis [1], Serena Faggiano [3,4], Samanta Raboni [3,4], Alessandra Gritti [5,6], Elisa Pianta [5], Valentina Pirovano [5], Giorgio Abbiati [5], Gloria Modafferi [7], Barbara Pioselli [7], Stefano Bruno [1,3], Barbara Campanini [1,3], Stefano Bettati [1,2,4,*] and Luca Ronda [1,2,4,*]

[1] Biopharmanet_TEC, University of Parma, Parco Area delle Scienze, 27/A, 43124 Parma, Italy; stefania.bova@unipr.it (S.B.); ilaria.denardis@unipr.it (I.D.N.); stefano.bruno@unipr.it (S.B.); barbara.campanini@unipr.it (B.C.)
[2] Department of Medicine and Surgery, University of Parma, Via Volturno, 39, 43125 Parma, Italy; marialaura.marchetti@unipr.it (M.M.)
[3] Department of Food and Drug, University of Parma, Parco Area delle Scienze, 27/A, 43124 Parma, Italy;
serena.faggiano@unipr.it (S.F.); samanta.raboni@unipr.it (S.R.)
[4] Institute of Biophysics, National Research Council, Via Giuseppe Moruzzi, 1, 56124 Pisa, Italy
[5] Department of Pharmaceutical Sciences, General and Organic Chemistry Section "A. Marchesini", University of Milan, Via Golgi 19, 20133 Milano, Italy; alessandra.gritti@unimi.it (A.G.);
elisa.pianta@unimi.it (E.P.); valentina.pirovano@unimi.it (V.P.); giorgio.abbiati@unimi.it (G.A.)
[6] Department of Chemistry, University of Milan, Via Golgi 19, 20133 Milano, Italy
[7] Chiesi Farmaceutici S.p.A., Global Research and Preclinical Development Area, Largo Belloli, 11/A,
43122 Parma, Italy; g.modafferi@chiesi.com (G.M.); b.pioselli@chiesi.com (B.P.)
* Correspondence: stefano.bettati@unipr.it (S.B.); luca.ronda@unipr.it (L.R.)

Experimental

All the reactions, that involve the use of reagents sensitive to oxygen or hydrolysis, were carried out under an inert atmosphere. The glassware was previously dried in an oven a 110 °C and set with cycles of vacuum and nitrogen. Syringes, used to transfer reagents and solvents, were previously set under a nitrogen atmosphere. Solvents and dry solvents were purchased from commercial suppliers. 2,4-Dihydroxybenzaldehyde, glicydol and w-bromo-ethyl esters **10a-c** were purchased from commercial suppliers. Ethyl 9-bromononanoate **10d** [24,25] and LDM **5** [8] were synthetized in our lab following procedures in the literature. The chromatographic column purifications were conducted by flash technique, using silica gel *Merck Grade* 9385 60Å (230-400 mesh). For thin-layer chromatography (TLC), silica gel 60778-25EA *FLUKA* thin-layer plates were employed. ^{1}H NMR analyses were performed with a Varian-Gemini 300 or a Brucker 400 Avance spectrometer at room temperature, at 300 or 400 MHz. The coupling constants (J) are expressed in Hertz (Hz) and the chemical shift (δ) in ppm. ^{13}C NMR analyses were performed with the same instruments, respectively, at 75.45 or 101 MHz; APT sequences were used to distinguish the methane and methyl carbon signals from those arising from methylene and quaternary carbon atoms.

LDMs Synthesis and Characterization

Ethyl 3-(4-formyl-3-hydroxyphenoxy)propanoate 11a

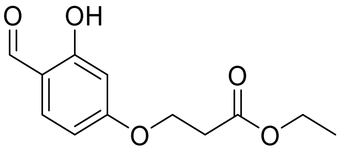

To a stirred solution of 2,4-dihydroxybenzaldehyde (500 mg, 3.62 mmol), Ag_2O (1.06 g, 4.60 mmol) and potassium iodide (60 mg, 0.36 mmol) in $CHCl_3$ (18 mL) at room temperature, ethyl 3-bromopropanoate (1.95 g, 10.80 mmol) was added and the mixture was heated ad reflux for 72 h. Then, another equivalent of ethyl 3-bromopropanoate **10a** was added and the mixture was heated for 48 h. The crude was filtered over a pad of celite and concentrated under vacuum. The mixture was purified by flash chromatography over silica gel (SiO_2, Hex/EtOAc = 5:1) to yield ethyl 3-(4-formyl-3-hydroxyphenoxy)propanoate **11a** (200 mg, 26%) as a white solid. ^1H NMR (300 MHz, $CDCl_3$): 11.44 (s, 1H), 9.71 (d, *J* = 0.6 Hz, 1H), 7.42 (d, *J* = 8.7 Hz, 1H), 6.53 (dd, *J* = 8.7, 2.3 Hz 1H), 6.43 (d, *J* = 2.3 Hz, 1H), 4.29 (t, *J* = 6.3 Hz, 2H), 4.19 (q, *J* = 7.1 Hz, 2H), 2.80 (t, *J* = 6.3 Hz, 2H), 1.27 (t, *J* = 7.1 Hz, 3H). ^{13}C NMR (75 MHz, $CDCl_3$): 194.41 (CH), 170.50 (C), 165.68 (C), 164.40 (C), 135.28 (CH), 115.34 (C), 108.56 (CH), 101.31 (CH), 63.85 (CH_2), 60.91 (CH_2), 34.23 (CH_2), 14.17 (CH_3).

3-(4-formyl-3-hydroxyphenoxy)propanoic acid 12a

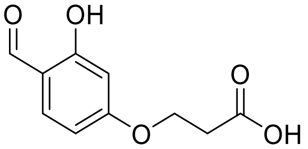

In a round-bottom flask, ethyl 3-(4-formyl-3-hydroxyphenoxy)propanoate **11a** (280 mg, 1.17 mmol) and trifluoroacetic acid (533 g, 4.68 mmol) were dissolved in water (6 mL). The reaction mixture was stirred at 90 °C for 7 h. Then, the mixture was extracted with EtOAc. The combined organic layers were dried over Na_2SO_4, filtered, and concentrated under reduced pressure to yield 3-(4-formyl-3-hydroxyphenoxy)propanoic acid **11b** (239 mg, 97%) as a white solid. ^1H NMR (300 MHz, DMSO): 12.38 (bs, 1H), 11.01 (bs, 1H), 9.99 (s, 1H), 7.59 (d, *J* = 8.7 Hz, 1H), 6.53 (dd, *J* = 8.7, 2.3 Hz, 1H), 6.46 (d, *J* = 2.3 Hz, 1H), 4.19 (t, *J* = 5.9 Hz, 2H), 2.69 (t, *J* = 6.0 Hz, 2H). ^{13}C NMR (75 MHz, DMSO): 191.40 (CH), 172.50 (C), 165.40 (C), 163.45 (C), 132.58 (CH), 116.72 (C), 108.05 (CH), 101.64 (CH), 64.55 (CH_2), 34.22 (CH_2).

Oxiran-2-ylmethyl 3-(4-formyl-3-hydroxyphenoxy)propanoate 6

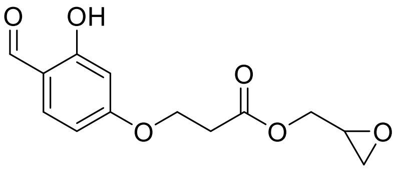

In a round-bottom flask, 3-(4-formyl-3-hydroxyphenoxy)propanoic acid **12a** (200 mg, 0.91 mmol), DCC (204 mg, 1.00 mmol) and DMAP (33 mg, 0.27 mmol) were dissolved in anhydrous CH_2Cl_2/DMF = 4 : 1 (9 mL) and stirred for 0.5 h at 0 °C. Then, glycidol (133 mg, 1.81 mmol) was added and stirred at room temperature for 22 h. The reaction mixture was filtered over a pad of celite and the filtrate was concentrated under reduced pressure. The residue was purified by flash chromatography over silica gel (SiO_2, CH_2Cl_2/EtOAc from 99:1 to 95:5) to yield oxiran-2-ylmethyl 4-(4-formyl-3-hydroxyphenoxy)butanoate **6** (75 mg, 30%) as a white solid. ^1H NMR (300 MHz, $CDCl_3$): 11.45 (d, *J* = 0.4 Hz, 1H), 9.72 (d, *J* = 0.5 Hz, 1H), 7.43 (d, *J* = 8.7 Hz, 1H), 6.53 (dd, *J* = 8.7, 2.4 Hz, 1H), 6.43 (s, 1H), 4.50 (dd, *J* = 12.3, 2.9 Hz, 1H), 4.31 (t, *J* = 6.2 Hz, 2H), 3.97 (dd, *J* = 12.3, 6.4 Hz, 1H), 3.23 (m, 1H), 2.95 – 2.82 (m, 3H), 2.66 (dd, *J* = 4.8, 2.6 Hz, 1H). ^{13}C NMR (75 MHz, $CDCl_3$): 194.47 (CH), 170.28 (C), 165.55 (C), 164.40 (C), 135.33 (CH), 115.38 (C), 108.54 (CH), 101.30 (CH), 65.40 (CH_2), 63.61 (CH_2), 49.21 (CH), 44.63 (CH_2), 33.99 (CH_2).

Ethyl 5-(4-formyl-3-hydroxyphenoxy)pentanoate 11b

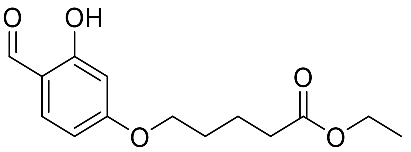

In a round-bottom flask (F1), 2,4-dihydroxybenzaldehyde (1 g, 7.24 mmol) and K_2CO_3 (1 g, 7.24 mmol) were dissolved in acetone (7.24 mL) and refluxed for 2 h under nitrogen atmosphere. Simultaneously, in another flask (F2), ethyl 5-bromopentanoate **10b** (1.51 g, 7.24 mmol) and sodium iodide (1.19 g, 7.96 mmol) in acetone (8 mL) were stirred at room temperature for 2 h under nitrogen atmosphere. Then, the solution in F2 was transferred to F1 and refluxed for another 24 h. The solvent was removed under reduced pressure and the crude was resuspended in water and extracted with EtOAc. The combined organic layers were dried over Na_2SO_4, filtered, and concentrated under reduced pressure. The crude material was purified by flash chromatography over silica gel (SiO_2,

Hex/EtOAc from 9:1 to 4:1) to yield ethyl 5-(4-formyl-3-hydroxyphenoxy)pentanoate **11b** (860 mg, 45%) as a white solid. ^{1}H NMR (300 MHz, $CDCl_3$): 11.47 (s, 1H), 9.70 (d, *J* = 0.6 Hz, 1H), 7.41 (d, *J* = 8.7 Hz, 1H), 6.51 (dd, *J* = 8.7, 2.3 Hz, 1H), 6.39 (d, *J* = 2.3 Hz, 1H), 4.13 (q, *J* = 7.1 Hz, 2H), 4.05 – 3.98 (m, 2H), 2.38 (t, *J* = 6.9 Hz, 2H), 1.98 – 1.73 (m, 4H), 1.25 (t, *J* = 7.1 Hz, 3H). ^{13}C NMR (75 MHz, $CDCl_3$): 194.34 (CH), 173.27 (C), 166.19 (C), 164.48 (C), 135.23 (CH), 115.09 (C), 108.68 (CH), 101.05 (CH), 67.97 (CH_2), 60.38 (CH_2), 33.80 (CH_2), 28.33 (CH_2), 21.49 (CH_2), 14.24 (CH_3).

5-(4-formyl-3-hydroxyphenoxy)pentanoic acid 12b

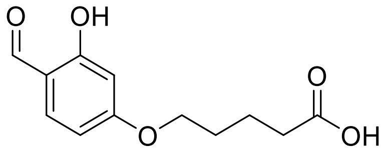

In a round-bottom flask (F1), ethyl 5-(4-formyl-3-hydroxyphenoxy)pentanoate **11b** (800 mg, 3 mmol) and trifluoroacetic acid (1.37 g, 12 mmol) were dissolved in water (15 mL). The reaction mixture was stirred at 90 °C for 7 h. Then, the mixture was extracted with EtOAc. The combined organic layers were dried over Na_2SO_4, filtered, and concentrated under reduced pressure to yield 5-(4-formyl-3-hydroxyphenoxy)pentanoic acid **11b** (574 mg, 80%) as a white solid. ^{1}H NMR (400 MHz, $CDCl_3$): 11.48 (s, 1H), 9.72 (s, 1H), 7.44 (d, *J* = 8.7 Hz, 1H), 6.54 (dd, *J* = 8.7, 2.3 Hz, 1H), 6.42 (d, *J* = 2.3 Hz, 1H), 4.05 (t, *J* = 5.7 Hz, 2H), 2.47 (t, *J* = 6.8 Hz, 2H), 1.95 – 1.76 (m, 4H). ^{13}C NMR (101 MHz, $CDCl_3$): 194.39 (CH), 179.34 (C), 166.18 (C), 164.51 (C), 135.29 (CH), 115.15 (C), 108.71 (CH), 101.11 (CH), 67.93 (CH_2), 33.50 (CH_2), 28.26 (CH_2), 21.27 (CH_2).

Oxiran-2-ylmethyl 5-(4-formyl-3-hydroxyphenoxy)pentanoate 7

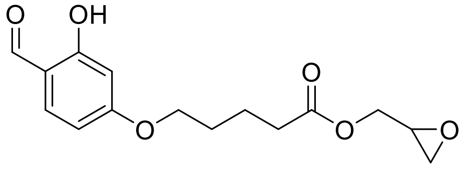

In a round-bottom flask, 5-(4-formyl-3-hydroxyphenoxy)pentanoic acid (500 mg, 2.10 mmol), EDC (805 mg, 4.2 mmol) and DMAP (77 mg, 0.63 mmol) were dissolved in anhydrous CH_2Cl_2/DMF = 4 : 1 (21 mL) and stirred for 0.5 h at 0 °C. Then, glycidol (311 mg, 4.2 mmol) was added and stirred at room temperature for 22 h. The reaction mixture was extracted with CH_2Cl_2 and water. The combined organic layers were dried over Na_2SO_4, filtered, and concentrated under reduced pressure. The residue was purified by flash chromatography over silica gel (SiO_2, Hex/EtOAc = 2:1) to yield Oxiran-2-ylmethyl 5-(4-formyl-3-hydroxyphenoxy)pentanoate **7** (140 mg, 23%) as a white solid. ^{1}H NMR (400 MHz, $CDCl_3$): 11.48 (s, 1H), 9.73 (s, 1H), 7.44 (d, *J* = 8.7 Hz, 1H), 6.54 (dd, *J* = 8.7, 2.3 Hz, 1H), 6.42 (d, *J* = 2.3 Hz, 1H), 4.46 (dd, *J* = 12.3, 3.0 Hz, 1H), 4.05 (t, *J* = 3.7 Hz, 2H), 3.94 (dd, *J* = 12.3, 6.4 Hz, 1H), 3.23 (ddt, *J* = 6.8, 4.1, 2.8 Hz, 1H), 2.87 (dd, *J* = 4.9, 4.1 Hz, 1H), 2.67 (dd, *J* = 4.9, 2.6 Hz, 1H), 2.54 – 2.42 (m, 2H), 1.93 – 1.80 (m, 4H). ^{13}C NMR (101 MHz, $CDCl_3$): 194.35 (CH), 172.94 (C), 166.18 (C), 164.51 (C), 135.26 (CH), 115.16 (C), 108.69 (CH), 101.12 (CH), 67.93 (CH_2), 64.97 (CH_2), 49.33 (CH), 44.65 (CH_2), 33.54 (CH_2), 28.32 (CH_2), 21.46 (CH_2).

Ethyl 7-(4-formyl-3-hydroxyphenoxy)heptanoate 11c

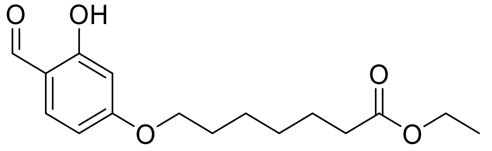

In a round-bottom flask (F1), 2,4-dihydroxybenzaldehyde (1 g, 7.24 mmol) and K_2CO_3 (1 g, 7.24 mmol) were dissolved in acetone (7.24 mL) and refluxed for 2 h under nitrogen atmosphere. Simultaneously, in another flask (F2), ethyl 7-bromoheptanoate **10c** (1.41 mL, 7.24 mmol) and sodium iodide (1.19 g, 7.96 mmol) in acetone (8 mL) were stirred at room temperature for 2 h under nitrogen atmosphere. Then, the solution in F2 was transferred to F1 and refluxed for 24 h. The solvent was removed under reduced pressure and the crude was resuspended in water and extracted with EtOAc. The combined organic layers were dried over Na_2SO_4, filtered, and concentrated under reduced pressure. The crude material was purified by flash chromatography over silica gel (SiO_2, Hex/EtOAc from 95:5 to 9:1 + 1% DCM) to give ethyl 7-(4-formyl-3-

hydroxyphenoxy)heptanoate **11c** (1.38g, 65%) as a white solid. ^{1}H NMR (300 MHz, $CDCl_3$): 11.46 (s, 1H), 9.69 (s, 1H), 7.40 (d, *J* = 8.6 Hz, 1H), 6.51 (dd, *J* = 8.7, 2.3 Hz, 1H), 6.39 (d, *J* = 2.3 Hz, 1H), 4.11 (q, *J* = 7.2 Hz, 2H), 3.98 (t, *J* = 6.4 Hz, 2H), 2.30 (t, *J* = 7.5 Hz, 2H), 1.85 – 1.72 (m, 2H), 1.65 (p, *J* = 7.3 Hz, 2H), 1.54 – 1.31 (m, 4H), 1.24 (t, *J* = 7.1 Hz, 3H). ^{13}C NMR (75 MHz, $CDCl_3$): 194.28 (CH), 173.66 (C), 166.36 (C), 164.49 (C), 135.20 (CH), 115.03 (C), 108.71 (CH), 101.05 (CH), 68.38 (CH_2), 60.21 (CH_2), 34.19 (CH_2), 28.75 (CH_2), 28.71 (CH_2), 25.60 (CH_2), 24.78 (CH_2), 14.23 (CH_3).

7-(4-formyl-3-hydroxyphenoxy)heptanoic acid 12c

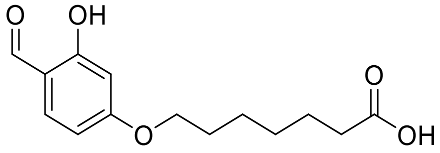

In a round-bottom flask, ethyl 7-(4-formyl-3-hydroxyphenoxy)heptanoate **11c** (1.3 g, 4.41 mmol) and trifluoroacetic acid (1.35 mL, 17.65 mmol) were dissolved in water (22.6 mL). The reaction mixture was stirred at 90 °C for 13 h. Then, the mixture was extracted with EtOAc. The combined organic layers were washed with brine and dried over Na_2SO_4, filtered, and concentrated under reduced pressure to give 3-(4-formyl-3-hydroxyphenoxy)propanoic acid **12c** (1.17 g, > 99%) as a white solid. ^{1}H NMR (300 MHz, $CDCl_3$): 11.47 (s, 1H), 9.70 (s, 1H), 7.41 (d, *J* = 8.7 Hz, 1H), 6.56 – 6.49 (m, 1H), 6.40 (d, *J* = 2.3 Hz, 1H), 4.00 (t, *J* = 6.4 Hz, 2H), 2.37 (t, *J* = 7.4 Hz, 2H), 1.86 – 1.75 (m, 2H), 1.67 (p, *J* = 7.5 Hz, 2H), 1.55 – 1.36 (m, 4H). ^{13}C NMR (75 MHz, $CDCl_3$): 194.79 (CH), 179.58 (C), 166.38(C), 164.51 (C), 135.40 (CH), 115.04 (C), 108.76 (CH), 101.06 (CH), 68.36 (CH_2), 33.84 (CH_2), 28.69 (CH_2), 28.65 (CH_2), 25.60 (CH_2), 24.49 (CH_2).

Oxiran-2-ylmethyl 7-(4-formyl-3-hydroxyphenoxy)heptanoate 8

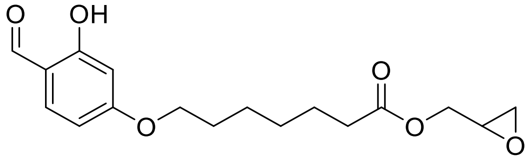

In a round-bottom flask, 3-(4-formyl-3-hydroxyphenoxy)heptanoic acid **4d** (1 g, 3.75 mmol) were dissolved in anhydrous CH_2Cl_2 (30 mL, 0.125 M) and anhydrous DMF (3 mL, 0.4 M). EDC (1.43 g, 7.5 mmol) and DMAP (138 mg, 1.13 mmol) were added; the mixture was stirred at 0 °C under N_2 for 30 minutes. Then, glycidol (190 µl, 2.84 mmol) was added and the reaction mixture was stirred at room temperature for 22 h. Next, H_2O was added, and the crude was extracted with DCM. The organic layers were dried over Na_2SO_4 and concentrated under reduced pressure. The residue was purified by flash chromatography over silica gel (SiO_2, Hex/EtOAc from 3:1 to 1:1) to give oxiran-2-ylmethyl 7-(4-formyl-3-hydroxyphenoxy)heptanoate **6d** (362mg, 30%) as a white solid. ^{1}H NMR (400 MHz, $CDCl_3$): 11.48 (s, 1H), 9.72 (s, 1H), 7.43 (d, *J* = 8.6 Hz, 1H), 6.53 (dd, *J* = 8.7, 2.3 Hz, 1H), 6.42 (d, *J* = 2.3 Hz, 1H), 4.44 (dd, *J* = 12.3, 3.0 Hz, 1H), 4.02 (t, *J* = 6.4 Hz, 2H), 3.92 (dd, *J* = 12.3, 6.4 Hz, 1H), 3.22 (m, 1H), 2.86 (dd, *J* = 4.9, 4.1 Hz, 1H), 2.66 (dd, *J* = 4.9, 2.6 Hz, 1H), 2.39 (t, *J* = 7.5 Hz, 2H), 1.88 – 1.76 (m, 2H), 1.75 – 1.64 (m, 2H), 1.56 – 1.36 (m, 4H). ^{13}C NMR (101 MHz, $CDCl_3$): 194.31 (CH), 173.32 (C), 166.38 (C), 164.52 (C), 135.23 (CH), 115.07 (C), 108.74 (CH), 101.08 (CH), 68.37 (CH_2), 64.84 (CH_2), 49.37 (CH), 44.64 (CH_2), 33.91 (CH_2), 28.72 (CH_2), 28,68 (CH_2), 25.61 (CH_2), 24.70 (CH_2).

Ethyl 9-(4-formyl-3-hydroxyphenoxy)nonanoate 11d

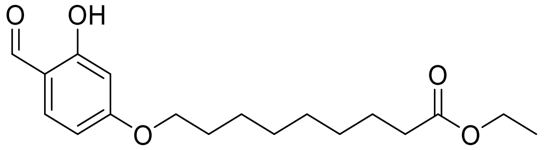

In a round-bottom flask (F1), 2,4-dihydroxybenzaldehyde (520 mg, 3.77 mmol) and K_2CO_3 (520 mg, 4.15 mmol) were dissolved in acetone (4 mL) and refluxed for 2 h under nitrogen atmosphere. Simultaneously, in another flask (F2), ethyl 8-bromooctanoate **10d** (1 g, 3.77 mmol) and sodium iodide (809 mg, 4.15 mmol) in acetone (4 mL) were stirred at room temperature for 2 h under nitrogen atmosphere. Then, the solution in F2 was transferred to F1 and refluxed for another 13 h. The solvent was removed under reduced pressure and the crude was resuspended in water and extracted with EtOAc. The

combined organic layers were dried over Na_2SO_4, filtered, and concentrated under reduced pressure. The crude material was purified by flash chromatography over silica gel (SiO_2, Hex/EtOAc = 3:1) to yield ethyl 7-(4-formyl-3-hydroxyphenoxy)nonanoate **11d** (607 mg, 54%) as a white solid. ^{1}H NMR (300 MHz, $CDCl_3$): 11.47 (s, 1H), 9.70 (s, 1H), 7.41 (d, *J* = 8.6 Hz, 1H), 6.52 (dd, *J* = 8.7, 2.3 Hz, 1H), 6.40 (d, *J* = 2.3 Hz, 1H), 4.12 (q, *J* = 7.1 Hz, 2H), 4.00 (t, *J* = 6.6 Hz, 2H), 2.29 (t, *J* = 7.5 Hz, 2H), 1.85 – 1.73 (m, 2H), 1.62 (t, *J* = 7.0 Hz, 2H), 1.51 – 1.39 (m, 2H), 1.34 (d, *J* = 4.3 Hz, 6H), 1.25 (t, *J* = 7.1 Hz, 3H). ^{13}C NMR (101 MHz, $CDCl_3$): 194.29 (CH), 173.83 (C), 166.45 (C), 164.54 (C), 135.21 (CH), 115.04 (C), 108.76 (CH), 101.08 (CH), 68.55 (CH_2), 60.18 (CH_2), 34.34 (CH_2), 29.13 (CH_2), 29.09 (CH_2), 29.03 (CH_2), 28.89 (CH_2), 25.86 (CH_2), 24.92 (CH_2), 14.26 (CH_3).

9-(4-formyl-3-hydroxyphenoxy)nonanoic acid 12d

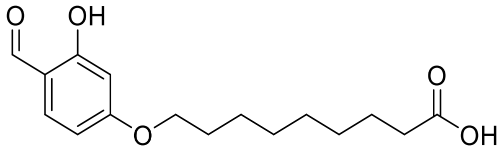

In a round-bottom flask, ethyl 7-(4-formyl-3-hydroxyphenoxy)nonanoate **11d** (640 mg, 1.99 mmol) and trifluoroacetic acid (608 µl, 7.95 mmol) were dissolved in water (10 mL) under nitrogen atmosphere. The reaction mixture was stirred at 90 °C for 13 h. Then, the mixture was extracted with EtOAc. The combined organic layers were washed with brine and were dried over Na_2SO_4, filtered, and concentrated under reduced pressure to obtain 3-(4-formyl-3-hydroxyphenoxy)nonanoic acid **12d** (433 mg, 74%) as a white solid. ^{1}H NMR (300 MHz, $CDCl_3$): 11.46 (s, 1H), 9.69 (s, 1H), 7.40 (d, *J* = 8.6 Hz, 1H), 6.51 (dd, *J* = 8.6, 2.3 Hz, 1H), 6.40 (d, *J* = 2.9 Hz, 1H), 3.99 (t, *J* = 6.5 Hz, 2H), 2.35 (t, *J* = 7.5 Hz, 2H), 1.84 – 1.72 (m, 2H), 1.63 (t, *J* = 7.5 Hz, 2H), 1.51 – 1.40 (m, 2H), 1.34 (s, 6H). ^{13}C NMR (75 MHz, $CDCl_3$): 194.32 (CH), 179.90 (C), 166.44 (C), 164.51 (C), 135.22 (CH), 115.00 (C), 108.77 (CH), 101.06 (CH), 68.52 (CH_2), 34.32 (CH_2), 29.07 (CH_2), 29.03 (CH_2), 28.91 (CH_2), 28.85 (CH_2), 25.82 (CH_2), 24.59 (CH_2).

Oxiran-2-ylmethyl 9-(4-formyl-3-hydroxyphenoxy)nonanoate 9

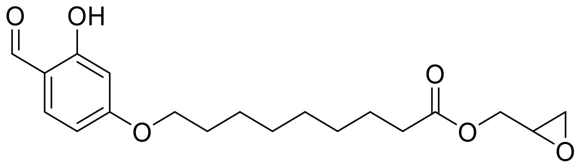

In a round-bottom flask, 3-(4-formyl-3-hydroxyphenoxy)propanoic acid **12d** (420 mg, 1.42 mmol) was dissolved in anhydrous CH_2Cl_2 (7.23 mL) and stirred at 0°C under N_2 atmosphere. EDC·HCl (327 mg, 2.84 mmol), DIEA (296 µl, 1.7 mmol) and DMAP (17 mg, 0.142 mmol) were added, and the reaction mixture was stirred 0 °C for 1 h. Then, glycidol (190 µl, 2.84 mmol) was added and the reaction mixture was stirred at room temperature for 20 h. It was quenched with H_2O washed with $NaHCO_3$ s.s and HCl. The organic layers were dried over Na_2SO_4 and concentrated under reduced pressure. The residue was purified by chromatography with Biotageâ Selekt (Biotage® HP-Sphere Flash Cartridge 10 g) to give oxiran-2-ylmethyl 9-(4-formyl-3-hydroxyphenoxy)nonanoate **6e** (20 mg, 4%) as a white solid. ^{1}H NMR (300 MHz, $CDCl_3$): 11.47 (s, 1H), 9.69 (s, 1H), 7.41 (d, *J* = 8.6 Hz, 1H), 6.51 (dd, *J* = 8.7, 2.3 Hz, 1H), 6.39 (d, *J* = 2.3 Hz, 1H), 4.42 (dd, *J* = 12.3, 3.0 Hz, 1H), 3.99 (t, *J* = 6.5 Hz, 2H), 3.89 (dd, *J* = 12.3, 6.4 Hz, 1H), 3.20 (m, 1H), 2.84 (dd, *J* = 4.9, 4.1 Hz, 1H), 2.64 (dd, *J* = 4.9, 2.6 Hz, 1H), 2.35 (t, *J* = 7.5 Hz, 2H), 1.85 – 1.70 (m, 2H), 1.68 – 1.60 (m, 2H), 1.50 – 1.30 (m, 8H). ^{13}C NMR (75 MHz, $CDCl_3$): 194.29 (CH), 173.46 (C), 166.42 (C), 164.50 (C), 135.20 (CH), 115.01 (C), 108.75 (CH), 101.05 (CH), 68.52 (CH_2), 64.78 (CH_2), 49.38 (CH), 44.63 (CH), 34.00 (CH_2), 29.09 (CH_2), 29.05 (CH_2), 28.97 (CH_2), 28.86 (CH_2), 25.83 (CH

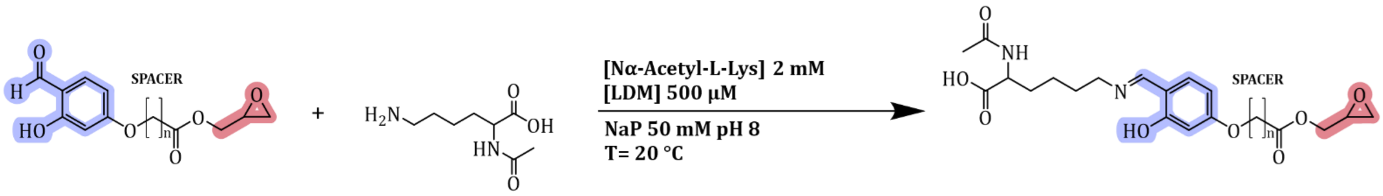

Scheme S1. Reaction of the LDM molecules with target free amino acid Nα-acetyl-L-lysine (NAL).

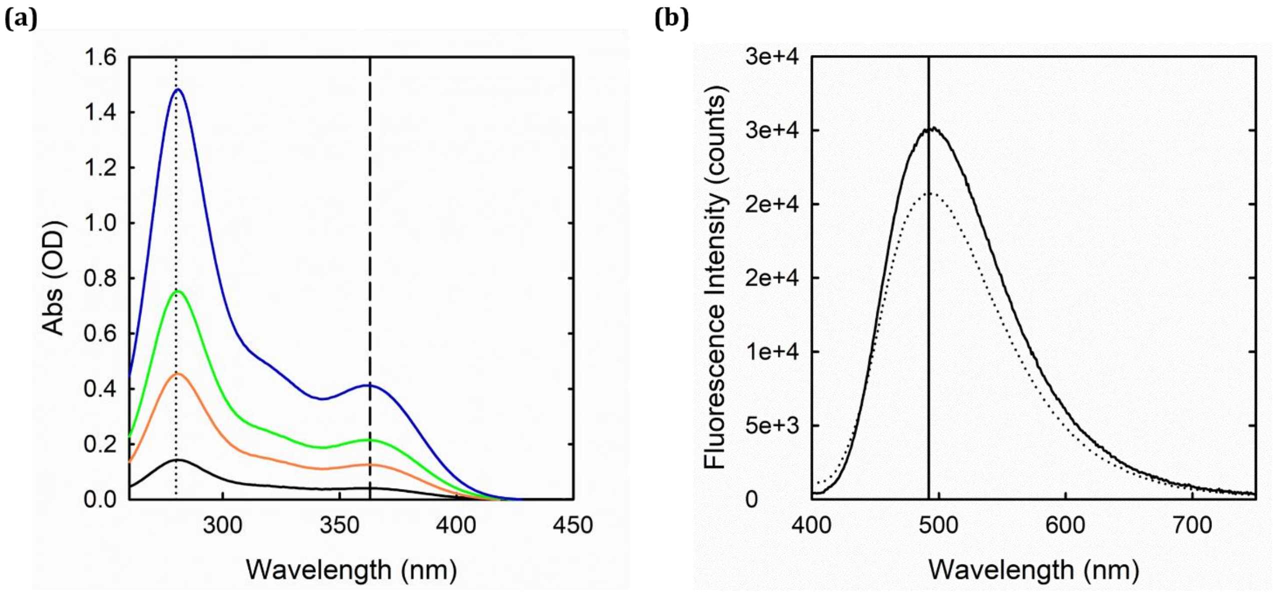

Figure S1. Spectroscopic characterization of LDM molecule 5. (**a**) Absorption spectra collected at different concentrations: 10 μM (black line), 30 μM (orange line), 50 μM (green line), and 100 μM (blue line). Reference lines are placed at 280 nm (dotted line) and 363 nm (dashed line). (**b**) Fluorescence emission spectra after excitation at 280 nm

(solid curve) or 363 nm (dotted curve). Reference line is placed at 490 nm (solid line).

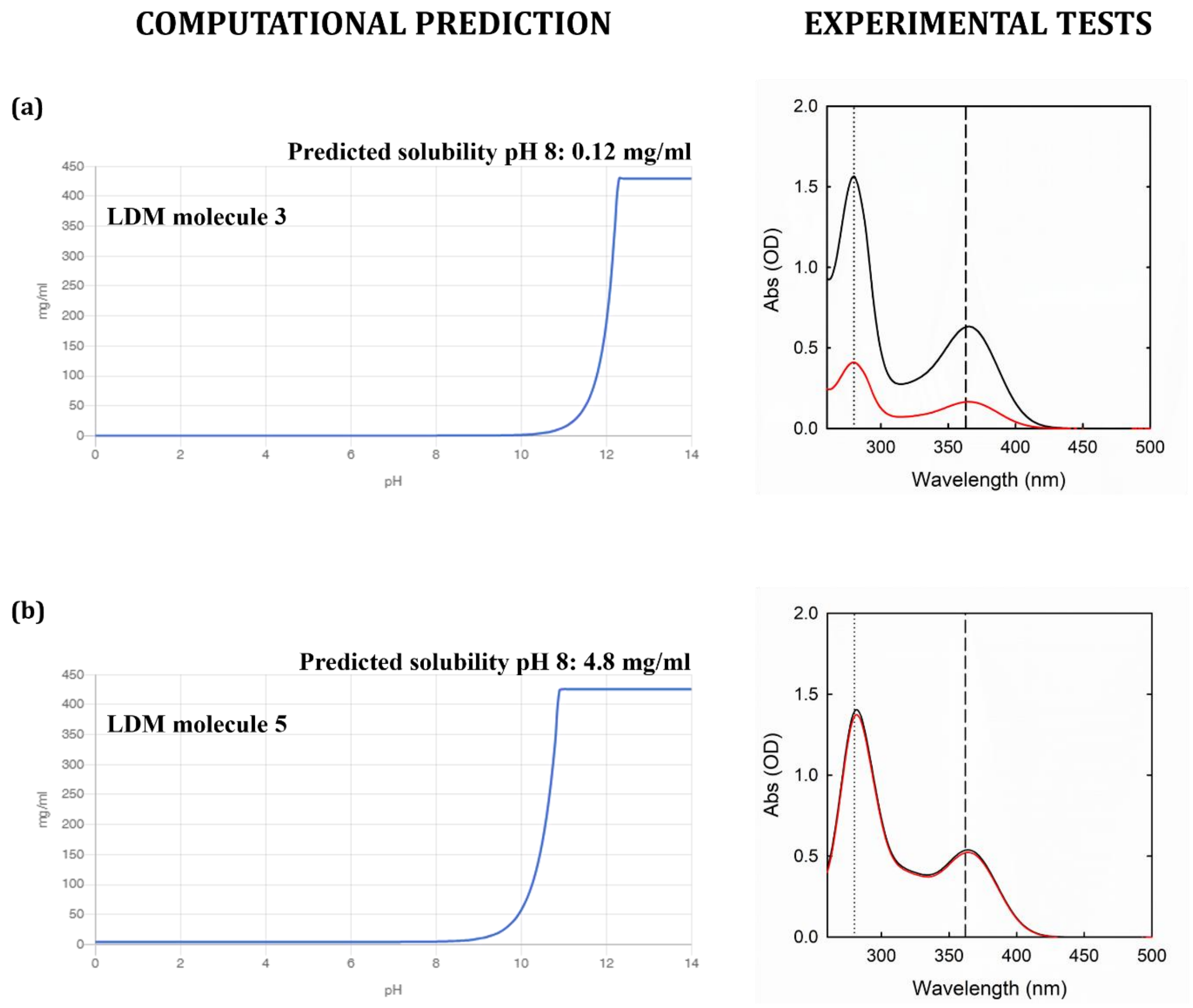

Figure S2. LDM molecule solubility characterization. Comparison between computational prediction (on the left) and experimental solubility tests (on the right) of 100 μ LDM molecules 3 (**a**) and 5 (**b**) at 20 °C. Spectra collected at time zero (black curve) and after centrifugation (red curve). Reference lines are placed at 280 (dotted line) and 363 nm (dashed line).

(a)

	HIS25	HIS77	HIS81	HIS139	HIS148	HIS169	HIS181	HIS199	HIS217
LYS3	45.5	24.67	20.11	43.11	34.96	35.81	30.78	20.39	38.11
LYS26	10.48	45.28	41.88	17.22	29.02	22.32	21.52	35.68	25.96
LYS41	36.77	22.3	26.05	29.45	21.73	23.12	23.13	20.67	18.85
LYS45	25.47	37.34	38.88	19.14	25.32	21.49	23.99	32.06	5.45
LYS52	11.12	46.46	46.52	7.72	26.41	19.59	23.81	39.17	14.38
LYS79	43.85	10.56	12.64	38.28	24.8	28.41	25	11.23	32.04
LYS85	37.92	14.23	13.82	32.94	21.37	23.7	19.91	9.53	27.6
LYS101	23.91	36.09	19.67	21.24	15.82	15.14	16.44	29.64	32.15
LYS107	15.17	36.97	33.16	17.89	20.94	15.91	13.03	27.3	27.78
LYS113	29.56	31.92	27.75	29.16	28.67	25.89	21.69	23.34	26.94
LYS126	10.18	42.08	38.65	15.82	24.74	18.44	17.32	32.49	26.76
LYS131	16.7	42	40.79	16.15	19.97	15.89	18.37	34.54	29.83
LYS140	14.33	44.03	44.32	10.05	20.65	15.4	20.65	37.3	23.3
LYS156	43.23	18.27	9.69	42.04	28	31.22	25.12	13.11	43.15
LYS158	34.54	27.01	19.67	34.64	25.52	26	19.5	18.03	39.22
LYS162	33.2	23.37	17.46	31.86	20.09	21.75	15.58	14.73	36.6
LYS166	29.93	27.91	26.8	23.27	10.84	12.95	11.58	21.45	30.93
LYS209	18.66	37.16	38.64	10	18.99	13.96	18.69	31.25	5.51
LYS214	27.13	47	48.96	21.64	32.72	28.17	32.02	41.99	10.66

Within the range
≤ 15.1 Å + 0.5 tolerance
Out of range
> 15.6 Å + 0.5 tolerance

(b)

	HIS25	HIS77	HIS81	HIS139	HIS148	HIS169	HIS181	HIS199	HIS217
LYS3									
LYS26	10.48								
LYS41									
LYS45									5.45
LYS52	11.12			7.72					14.38
LYS79		10.56	12.64					11.23	
LYS85		14.23	13.82					9.53	
LYS101						15.14			
LYS107	15.17						13.03		
LYS113									
LYS126	10.18								
LYS131									
LYS140	14.33			10.05		15.4			
LYS156			9.69					13.11	
LYS158									
LYS162							15.58	14.73	
LYS166					10.84	12.95	11.58		
LYS209				10		13.96			5.51
LYS214									10.66

Within the range and without steric hindrance
Within the range and with steric hindrance

Figure S3. Structure-based selection of GFP residues possibly involved in interaction with LDM molecules. Panel a: All distances below maximum LDM distances (15.5 Å) in green. Panel b: All distances below maximum LDM distances (15.5 Å) and without steric hindrance between the amino acid residues in green, or with steric hindrances in red.

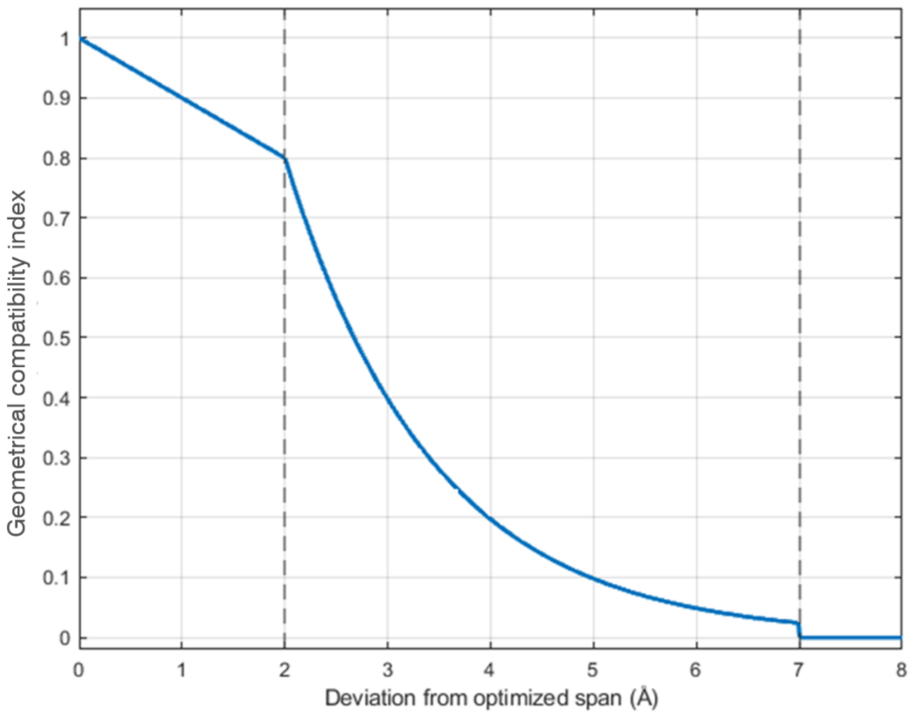

Figure S4 *Geometrical compatibility index. Geometrical compatibility index as a function of the difference between Lys-His distance and reactive groups on LDM molecule. The index reaches its maximum (1) when the inter-residue distance matches the molecular distance, decreases linearly within the first 2 Å of shortening, and then decays exponentially for shorter distances, approaching zero at 7 Å below the optimal span. Distances differences exceeding the optimized distance between reactive groups on LDM molecule are assigned a index of zero.*

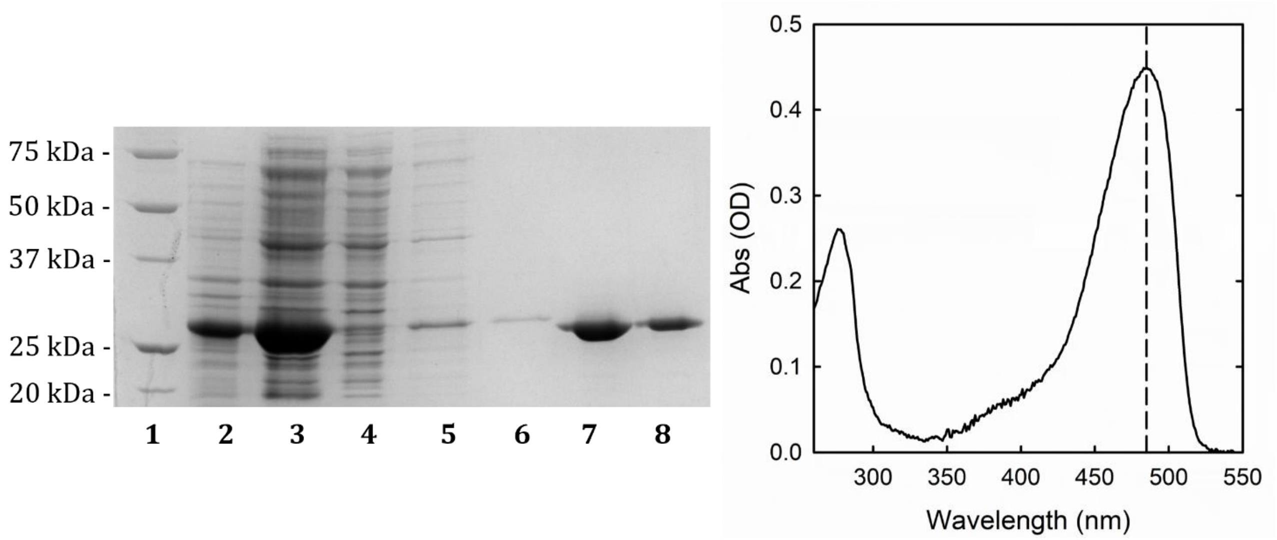

Figure S5. On the left, SDS-PAGE Tris-Glycine analysis of GFPmut2 purification. Lane 1: molecular weight marker; lane 2: insoluble fraction; lane 3: soluble fraction; lane 4: flow-through; lane 5-6: wash fractions, lane 7-8: elution fractions at different dilution. On the right, UV–vis absorption spectrum of the purified GFPmut2 solution in 50 mM Na phosphate buffer, pH 8. A reference line is placed at 485 nm (dashed line), corresponding to the chromophore's absorbance maximum at the given pH.

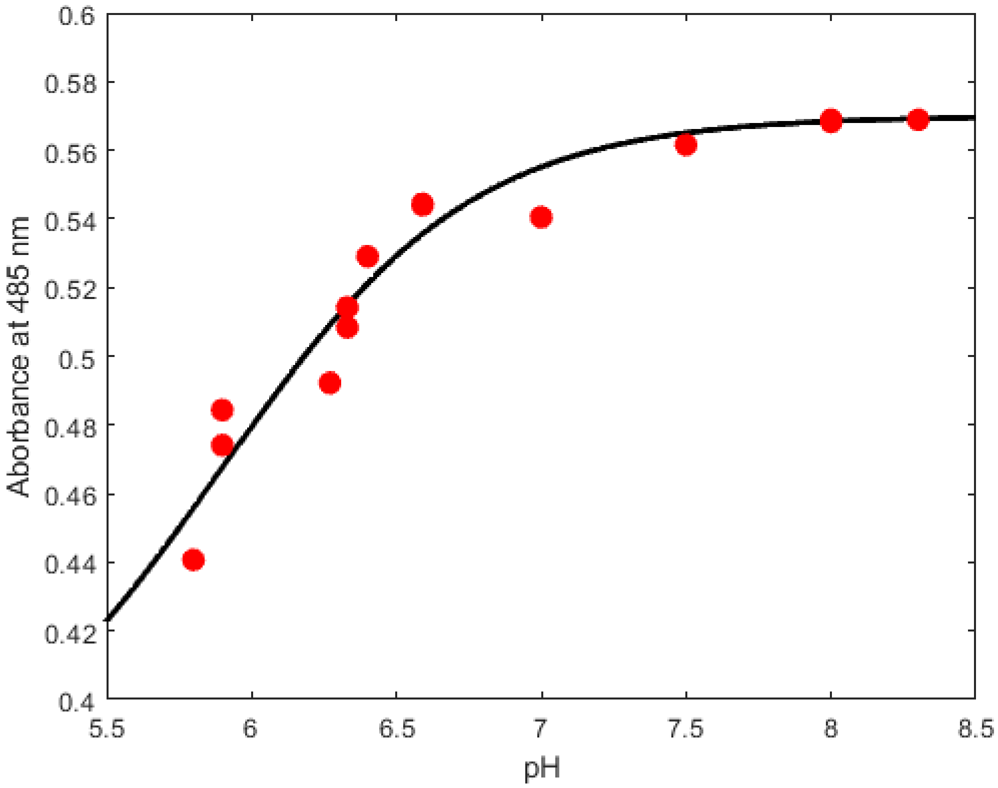

Figure S6. pH dependence of the absorbance at 485 nm of GFPmut2 modified with molecule 5 (closed red circles). The line represents the fitting to Equation 1 (Materials and Methods).

Table S1. Molar extinction coefficients of LDM molecules.

Molecules	$\varepsilon_{280\ nm}$	$\varepsilon_{363\ nm}$
**5**	14.832	4,124
**6**	12,697	3,853
**7**	14,196	3,731

Table S2. PropK$_a$ analysis: pK$_a$ values were averaged over the two crystallographic chains (A and B) and standard deviation was also calculated.

Residue	Averaged pKa	σ
**LYS3**	10.50	0.00
**LYS26**	10.15	0.00
**LYS41**	10.36	0.00
**LYS45**	10.22	0.21
**LYS52**	10.50	0.00
**LYS79**	9.65	0.01
**LYS85**	10.55	0.23
**LYS101**	10.29	0.00
**LYS107**	9.56	0.04
**LYS113**	10.15	0.00
**LYS126**	9.63	0.04
**LYS131**	10.29	0.00
**LYS140**	10.15	0.00
**LYS156**	10.50	0.00
**LYS158**	10.22	0.00
**LYS162**	10.50	0.00
**LYS166**	10.26	0.04
**LYS209**	10.38	0.23
**LYS214**	10.47	0.04
**HIS25**	7.42	0.02
**HIS77**	6.97	0.47
**HIS81**	7.09	0.07
**HIS139**	6.05	0.93
**HIS148**	-0.11	0.29
**HIS169**	1.94	0.23
**HIS181**	0.86	0.19
**HIS199**	4.22	0.43
**HIS217**	6.96	0.46
